# Search for translation arrest peptides encoded upstream of genes for components of protein localization pathways

**DOI:** 10.1093/nar/gkab024

**Published:** 2021-01-27

**Authors:** Karen Sakiyama, Naomi Shimokawa-Chiba, Keigo Fujiwara, Shinobu Chiba

**Affiliations:** Faculty of Life Sciences, Kyoto Sangyo University, Motoyama, Kamigamo, Kita-Ku, Kyoto 603-8555, Japan; Faculty of Life Sciences, Kyoto Sangyo University, Motoyama, Kamigamo, Kita-Ku, Kyoto 603-8555, Japan; Institute for Protein Dynamics, Kyoto Sangyo University, Japan; Faculty of Life Sciences, Kyoto Sangyo University, Motoyama, Kamigamo, Kita-Ku, Kyoto 603-8555, Japan; Institute for Protein Dynamics, Kyoto Sangyo University, Japan; Faculty of Life Sciences, Kyoto Sangyo University, Motoyama, Kamigamo, Kita-Ku, Kyoto 603-8555, Japan; Institute for Protein Dynamics, Kyoto Sangyo University, Japan

## Abstract

Regulatory nascent peptides participate in the regulation of cellular functions by the mechanisms involving regulated translation arrest. A class of them in bacteria, called monitoring substrates, feedback-regulates the expression of a specific component of protein localization machinery. Three monitoring substrates, SecM, MifM and VemP have previously been identified. Here, we attempt at identifying additional arrest peptides in bacteria. Our bioinformatic searches over more than 400 bacterial genomic sequences for proteins that have the common characteristic features shared by the known monitoring substrates and subsequent *in vitro* and *in vivo* characterization of the highlighted sequences allowed the identification of three arrest peptides termed ApcA, ApdA and ApdP. ApcA and ApdA homologs are conserved among a subset of actinobacteria, whereas ApdP has homologs in a subset of α-proteobacteria. We demonstrate that these arrest peptides, in their ribosome-tethered nascent states, inhibit peptidyl transfer. The elongation arrest occurs at a specific codon near the 3′ end of the coding region, in a manner depending on the amino acid sequence of the nascent chain. Interestingly, the arrest sequences of ApcA, ApdA and ApdP share a sequence R-A-P-G/P that is essential for the elongation arrest.

## INTRODUCTION

Regulatory nascent peptides occur in both bacterial and eukaryotic cells. They have a unique property of arresting their translation, either at the elongation or the termination step, for regulatory purposes (1,2). Their specific amino acid sequences, called arrest sequences, interact with the ribosomal components, typically near the peptidyl transferase center (PTC) and on the inner wall of the peptide exit tunnel, to elicit the temporary dysfunction of the ribosome (3). The regulatory nascent peptides brought about a new concept that nascent polypeptides could actively participate in the regulation of cellular functions instead of being mere intermediates of translation.

A class of regulatory nascent peptides in bacteria has been termed a monitoring substrate because it is a substrate of a protein localization machinery and, at the same time, monitors the activity of the machinery to feedback-control the expression of its key component (4,5). They include *Escherichia coli* SecM (6), *Bacillus subtilis* MifM (7) and *Vibrio alginolyticus* VemP (8), and share the following common features. First, they are encoded by an upstream open reading frame (ORF) of the regulatory target gene that encodes the key component of the protein localization machinery. Second, they have an arrest sequence near the C-termini. Thirdly, their N-terminal regions contain a topogenic sequence that is recognized by the localization machinery to be feedback-controlled. Finally, the elongation arrest is subject to cancellation by the action of the localization machinery that attempts to mobilize the nascent polypeptide into the translocation pathway. In the last property of a monitoring substrate, physical pulling forces generated by the engagement of the nascent polypeptide in translocation across or integration into the membrane are thought to disrupt the arrest sequence-ribosome interactions required for the translation arrest. The force-sensing nature of the elongation arrest provides the basis on which the monitoring substrates monitor the activity of the protein localization machinery to exert real-time feedback regulation of the expression of one of the machinery's key components (4,9–12).

Currently, the Sec protein translocation machinery and the YidC protein insertase are known as the targets of regulation by the respective monitoring substrates. The Sec system provides the major pathway, known as the Sec translocon, of protein translocation across and insertion into the membrane. In bacteria, membrane proteins SecY, SecE and SecG form a polypeptide-conducting channel (13), through which the substrate polypeptide chain moves to reach the extracytoplasmic (or periplasmic) side. SecA, an essential ATPase, plays a crucial role in driving the transmembrane polypeptide movement by pushing the substrate from the cytosol into the translocon channel (14). The SecDF (SecD–SecF) membrane protein complex enhances further translocation by binding/releasing the translocating chain from the extracytoplasmic side in a manner dependent on the transmembrane proton-motive force (15,16). In some bacteria, including *B. subtilis*, SecDF is produced as a single polypeptide from a single *secDF* gene (17). In *E. coli*, the gene *yajC* is co-transcribed with *secDF* and encodes a small membrane protein that interacts with SecDF (18), although its physiological function is unknown. SecB is a cytoplasmic protein conserved among proteobacteria (19) and serves as a chaperone to maintain newly synthesized preproteins translocation-competent (20). YidC is a bacterial member of the YidC/Oxa1/Alb3 family proteins, which facilitates membrane insertion of a class of membrane proteins that typically have one or two transmembrane segments and a small extracytoplasmic domain (21). Whereas the insertase function is executed independently of the Sec system, YidC also cooperates with the Sec machinery in supporting the biogenesis of more complex membrane proteins (22).

SecM is a monitoring substrate that regulates the expression of SecA. In *E. coli*, *secM* forms an operon with the downstream *secA* and comprises 170 codons (6). The *secM-secA* mRNA can form a stem-loop secondary structure at the *secM-secA* intergenic region, which sequesters the Shine-Dalgarno (SD) sequence required for the translation initiation of *secA* and consequently down-regulates the production of SecA. In the amino acid sequence of SecM, the N-terminal region contains the export signal sequence, which directs SecM to the Sec translocation pathway, whereas the C-terminal region contains the arrest sequence, which halts translation elongation of the SecM nascent chain (23). The elongation arrest, which takes place between Gly165 (situated at the ribosomal P-site) and Pro166 (at the A-site), causes a ribosome stalling in such a way to interfere with the formation of the stem-loop structure, thereby allowing active translation of *secA* (24). As already mentioned, the active engagement of the SecM nascent chain in the Sec translocation reaction leads to cancellation of the elongation arrest and consequent down-regulation of the *secA* translation. When the cellular activity of the Sec system declines, the duration of the elongation arrest prolongs, leading to the increased production of SecA (25).

Similar schemes of mechanism also operate for the regulation of YidC2 (YqjG) in *B. subtilis* (7) and SecDF2 in *Vibrio alginolyticus* (8). In the former case, *B. subtilis* induces YidC2, the secondary YidC homolog, in response to dysfunction of SpoIIIJ (YidC1), the primary YidC factor (26). MifM, encoded upstream of *yidC2*, plays a pivotal role in this regulation by undergoing elongation arrest that is sensitive to the YidC insertase action (7). VemP occurs in the *Vibrio* strain, a marine bacterium that contains sodium-dependent SecDF1 and proton-dependent SecDF2. SecDF2 is induced under low salinity conditions where SecDF1 cannot function. VemP, encoded upstream of *secDF2*, undergoes elongation arrest that is sensitive to the Sec export activity (8). Thus, VemP is crucial for the *secDF2* regulation and adaptation of this bacterium to low-salt water.

Structural studies of SecM and MifM revealed that these nascent chains adopt extended conformations in the exit tunnel of the ribosomes while the VemP nascent chain forms characteristic α-helices in the tunnel (27–30). As suggested from genetic studies (7–8,23,31), the cryo-EM structures revealed that the SecM, MifM and VemP nascent polypeptides contact with the ribosomes at multiple sites, including the constricted region of the exit tunnel and those proximal to the PTC. It is believed that these specific interactions between the nascent polypeptide and the ribosome lead to conformational anomalies of ribosomal components, including those preventing functional accommodation of amino acyl-tRNA to the A-site (28–30). An anomaly of the peptidyl-tRNA positioning at the P-site was also suggested for SecM (27), although it was not confirmed by a more recent cryo-EM study (28).

While these monitoring substrates regulate the respective target genes by common mechanisms involving regulated translation arrest, their arrest-provoking amino acid sequences lack any notable similarity (FXXXXWIXXXXGIRAGP for SecM, RIXXWIXXXXXMNXXXXDEED for MifM and HRIXGWKETNAMYVXLNXX for VemP; X indicates presumed space-fillers whose identities are unimportant for the arrest) (7–8,23,31–32). While SecA, SecDF and YidC are conserved among all bacterial species, these genes are not always accompanied by a monitoring substrate-encoding upstream ORF. The phylogenetic distribution of each monitoring substrate is narrow and limited to a specific lineage, which possesses only one of the three monitoring substrates (7–8,19). Thus, it is suggested that these regulatory factors have evolved lately and independently during evolution. Importantly, each of them should have given a selective advantage to the respective organism, as shown for the VemP’s contribution to the survival of marine bacteria upon exposure to pure water environments (8). It is conceivable that many more bacterial species exploit the ability of an arrest polypeptide of the monitoring substrate class to feedback regulate a protein localization factor in real-time.

Here, we describe our attempts to identify regulatory arrest peptides that may serve as a monitoring substrate of protein localization machinery. We combined bioinformatic searches over bacterial genomic sequences with *in vivo* and *in vitro* characterization of candidate ORFs and discovered three novel arrest peptides encoded by genes in front of the *sec*/*yidC* genes. We term the arrest peptide encoded upstream of *yidC* of actinobacteria ApcA, the arrest peptide encoded upstream of *secDF* of actinobacteria ApdA, and the arrest peptide encoded upstream of *secDF* of α-proteobacteria ApdP. We show that translation of these peptides is subject to elongation arrest at the peptidyl transfer step at specific P-site and A-site codons in a manner depending on the nascent chain's amino acid sequences, rather than mRNA nucleotide sequences, near their C-termini. Notably, they share an amino acid sequence motif near the arrest site that is crucial for the translation halt.

## MATERIALS AND METHODS

### Bioinformatics

**Figure 1.**
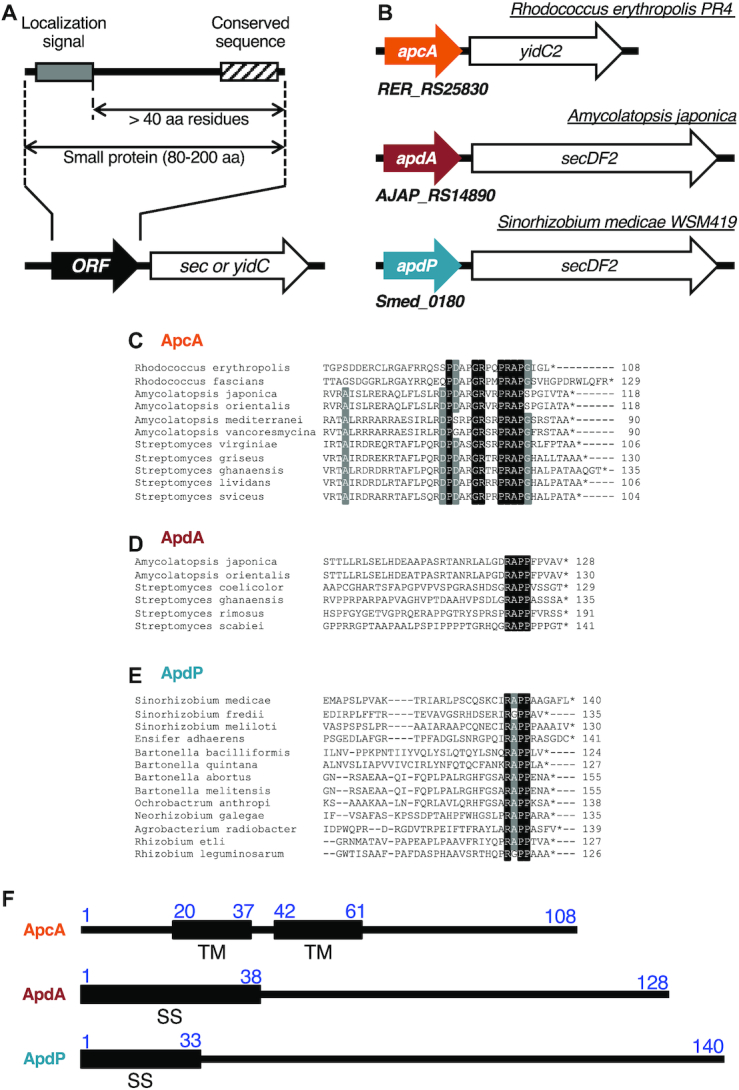
Bioinformatics approach for identification of translocation-related arrest peptides. (**A**) A schematic representation of the target ORFs. We searched for ORFs having the following properties: (1) located upstream of the *sec* or *yidC* genes, (2) encoding small proteins of 80–200 amino acids, (3) containing an N-terminal topogenic signal for extra-cytosolic localization, (4) having the following hydrophilic domain of at least 40 amino acid residues, and (5) showing subgroup (ApcA, ApdA and ApdP)-specific sequence conservations at the C-terminal regions. (**B**) Schematic representations of *apcA*, *apdA* and *apdP* with downstream sec/yidC genes. (**C–E**) Sequence alignment at the C-terminal ends among homologs of ApcA (C), ApdA (D) and ApdP (E). Black and gray backgrounds indicate completely and highly conserved residues, respectively. (**F**) Schematic representations of the topogenic signals of *R. erythropolis* ApcA, *A. japonica* ApdA and *S. medicae* ApdP with the amino acid numbering. ‘TM’ and ‘SS’ indicate transmembrane region and signal sequence, respectively.

In order to make a list of target bacterial species, we performed a keyword search using ‘*secY*’ on the NCBI gene website (https://www.ncbi.nlm.nih.gov/gene/) on an assumption that this gene is conserved among all bacterial species. The resulting list of the *secY* genes was downloaded, and the bacterial species were manually identified to extract the list of the target bacterial species (449 species; Supplementary Table S1). Then, we obtained the nucleotide sequences of individual *sec* or *yidC* genes and their surrounding regions of the chromosome using the NCBI gene website. We used the keywords, ‘*yidC*’, ‘*oxaA*’ and ‘*spoIIIJ*’ to search for *yidC* homologs and ‘*secD*’ and ‘*secDF*’ to search for *secD*, *secF* and *secDF*. To identify other *sec* genes, we used the keywords, ‘*secY*’, ‘*secE*’, ‘*secA*’ and ‘*secG*’, respectively. For instance, the keywords ‘*Bacillus subtilis*’ and ‘*secY*’ were used to extract *B. subtilis secY* sequence. Sometimes, we failed to identify *sec* (other than *secY*) or *yidC* homologs by our keyword search likely due to incomplete annotations. In such a case, we discontinued the search without attempting to resolve the problem. After identification of a *sec* or *yidC* gene, we carried out visual inspection of its upstream region of the chromosome to find out any upstream ORF that is oriented correctly and without a previous functional assignment. We then subjected the amino acid sequence of the ORF to the topogenic signal prediction programs, TMHMM v.2.0 (33) (http://www.cbs.dtu.dk/services/TMHMM/) and SignalP 4.0 (34) (http://www.cbs.dtu.dk/services/SignalP/). If the protein sequence contained a putative topogenic signal near the N-terminus, we then carried out the protein BLAST search (35) (https://blast.ncbi.nlm.nih.gov/Blast.cgi?PROGRAM=blastp&PAGE_TYPE=BlastSearch&LINK_LOC=blasthome) with the default setting to identify their homologs in other bacteria and, then, examined whether the C-terminal region is conserved among the homologs by sequence alignments. We note that the BLAST search allowed identification of ApcA homologs of the genus *Streptomyces*, whose gene annotations do not include a gene searchable by the keyword ‘*yidC*’. Clustal omega (36) (https://www.ebi.ac.uk/Tools/msa/clustalo/) was used to make multiple sequence alignments of ApcA and ApdP (Figure 1). The sequence alignment of ApdA in Figure 1D was done manually so as to align the RAPP sequence.

### Bacterial strains and plasmids

*B. subtilis* strains, plasmids and DNA oligonucleotides used in this study are listed in Supplementary Tables S2, S3 and S4, respectively. *E. coli* strains are listed in Supplementary Table S5. The *B. subtilis* strains were derivatives of PY79 (wildtype; (37)) and were constructed by transformation of the parent strains with plasmids listed in Supplementary Table S2. Plasmids were constructed by standard cloning methods, PCR, PrimeSTAR mutagenesis (Takara), Gibson assembly (38) and combinations of these methods using primers and template DNA as described in Supplementary Table S3. To randomize the A-site codons of *apcA*, *apdA* and *apdP*, randomized primers (Supplementary Tables S3 and S4) were used. Preparation of synthetic DNA for *apcA*, *apdA* and *apdP* were outsourced (ThermoFisher). Successful integration of a gene into the *B. subtilis* chromosome was accomplished by double crossing-over at the target loci. The resulting recombinant clones were checked for their antibiotic resistance markers, including the absence of those originally present on the plasmid backbone, and inactivation of the *amyE* target locus.

### Culture media and growth conditions

*B. subtilis* cells were cultured in LB medium. *E. coli* cells were cultured in LB medium supplemented with 100 μg/ml ampicillin. Cells were cultured at 37°C and withdrawn at an optical density at 600 nm (OD_600_) of 0.5–1.0 for western blotting or β-galactosidase activity assays.

### *In vitro* translation

*In vitro* translation was carried out using the *E. coli*-based coupled transcription-translation system composed of the purified components (PUREfrex 1.0; GeneFrontier), which we refer to *Ec* PURE system, and *Bs* (*B. subtilis*) hybrid PURE system, in which *B. subtilis* ribosome was combined with the translation components from *E. coli*, as described previously (12,39). For the latter, a preparation of the *B. subtilis* ribosomes was included at a final concentration of 1 μM. 2.5 U/μl of T7 RNA polymerase (Takara) was added further to the reaction mixture to reassure transcription. The DNA templates were prepared by PCR using appropriate primers and PCR templates (Supplementary Table S6). For Western blotting, we carried out the translation reaction at 37°C for 30 min. The reaction was stopped by adding three volumes of 1.3× SDS-PAGE loading buffer (167 mM Tris–HCl (pH 6.8), 2.7% (wt/vol) SDS, 20% (vol/vol) glycerol, 6.7 mM DTT, a trace amount of bromophenol blue). When indicated, samples were further treated with 0.2 mg/ml RNase A (Promega) at 37°C for 10 min immediately before electrophoresis.

### Western blotting

Samples in SDS-PAGE loading buffer were separated by 11% polyacrylamide gel prepared with WIDE RANGE Gel buffer (Nacalai Tasque) according to the manufacturer's instructions, transferred onto a PVDF membrane, and then subjected to immuno-detection using antibodies against GFP (A-6455; Invitrogen) or FLAG-tag (1E6; Wako). Images were obtained and analyzed using Amersham Imager 600 (GE Healthcare) luminoimager.

### β-Galactosidase assay

β-Galactosidase activity assay was carried out, as described previously (40). Briefly, 100 μl portions of the cultures were transferred to individual wells of 96-well plate, and OD_600_ was recorded. Cells in each well were then lysed with 50 μl of Y-PER reagent (Thermo Scientific) for 20 min at room temperature. After 30 μl of o-nitrophenyl-β-d-galactopyranoside (ONPG) in Z-buffer (60 mM Na_2_HPO_4_, 40 mM NaH_2_PO_4_, 10 mM KCl, 1mM MgSO_4_, 38 mM β-mercaptoethanol) was added to each well, OD_420_ and OD_550_ were measured every 5 min over 60 min at 28°C. Arbitrary units [AU] of β-galactosidase activity were calculated by the formula [(1000 × *V*_420_ – 1.3 × *V*_550_)/OD_600_], where *V*_420_ and *V*_550_ are the first-order rate constants, OD_420_/min and OD_550_/min, respectively.

### Toeprint analysis

*In vitro* translation reaction was primed with the *gfp-apcA*, *gfp-apdA* and *gfp-apdP* templates using *Bs* hybrid PURE system or the original PURE system and continued at 37°C for 20 min in the presence or absence of 0.1 mg/mL chloramphenicol. The templates for translation were prepared by PCR amplification using primers and PCR templates listed in Supplementary Table S6. The reaction was then mixed with the same volume of reverse transcription mixture (50 mM HEPES–KOH (pH7.6), 100 mM potassium glutamate, 2 mM spermidine, 13 mM magnesium acetate, 1 mM DTT, 2 μM fluorescently labeled DNA primer, 50 μM each of dNTPs, 10 units/μl ReverTra Ace purchased from Toyobo) and incubated further at 37°C for 15 min. The reaction mixture was diluted five-fold with the NTC buffer (Macherey-Nagel), and the reverse transcription products were purified using NucleoSpin Gel and PCR Clean-up kit (Macherey-Nagel). The elution of the reverse transcription products was done using 30 μl of HiDi formamide (Thermo Fisher). Samples were then mixed with 10 μl of GeneScan 500 LIZ dye size standard (Thermo), which serves as the internal size standard, that was beforehand diluted 10-fold with HiDi formamide, and heated at 96°C for 3 min just before capillary electrophoresis. The DNA primer for reverse transcription (5′-AACGACGGCCAGTGAATCCGTAATCATGGT-3′) was labeled with 6-carboxyfluorescein (6-FAM) at the 5′-end (Invitrogen). DNA templates commonly contained a sequence complementary to the reverse transcription primer at the 3′ region. Dideoxy DNA sequencing samples were prepared using Thermo Sequenase Dye Primer Manual Cycle Sequencing Kit (Thermo) according to the manufacture's instruction with some modifications as follows. The reaction was carried out using the same sets of templates and the 6-FAM-leveled primer as used for toeprinting. DNA polymerase reaction was carried out in the presence of 0.44 μM of the primer, 60 μM each of deoxynucleotides triphosphate (dATP, dCTP, dGTP and dGTP), and 0.6 μM of one of four dideoxynucleotide triphosphates (either ddATP, ddCTP, ddGTP or ddTTP). The sequence products were purified using Agencourt CleanSEQ (Beckman Coulter) and eluted with HiDi formamide. Then, 2 μl of GeneScan 500 LIZ dye size standard diluted ten-fold with HiDi formamide was added. Samples for toeprinting and dideoxy sequencing reactions were simultaneously subjected to capillary electrophoresis in the ABI PRISM 3130xl genetic analyzer (Applied Biosystems) and subsequently analyzed by HiTRACE-Web online software (http://hitrace.org/index.php/page/view/home) (41).

### Northern blotting

For northern blotting, samples containing peptidyl-tRNA were separated by 11% polyacrylamide gel prepared with WIDE RANGE Gel buffer, transferred onto a Hybond-N+ membrane (GE Healthcare), and hybridized with biotinylated oligodeoxynucleotide probes (purchased from Invitrogen) complementary to either tRNA^Gly^ or tRNA^Pro^ using NorthernMax kit (Ambion). Signals were detected with streptavidin-alkaline phosphatase using the BrightStar BioDetect kit (Ambion). Images were visualized and analyzed by LAS600 luminoimager (GE healthcare). The probes used were GlyT (5′-CAGCTTGGAAGGCTGAGGTAATAGC-3′), ProM (5′-CACTGGTCCCAAACCAGTTGC-3′), ProK (5′-CCTTCGTCCCGAACGAAGTGC-3′) and ProL (5′-CCCGACACCCCATGACGGTGC-3′). The GlyT probe was used for the detection of tRNA^Gly^, whereas the ProM and ProK probes were mixed and used for the detection of tRNA^Pro^ on the peptidyl-tRNA of the ApcA derivatives (Figure 6B). The ProL probe was used for the detection of tRNA^Pro^ that recognizes the CCC codon (Figure 6D and E).

## RESULTS

### Bioinformatic searches for bacterial open reading frames that meet the criteria of the monitoring substrates of the Sec or the YidC pathway

The known monitoring substrates, SecM, MifM and VemP are encoded upstream of genes for a component of protein localization machinery and have N-terminal protein localization signals and the C-terminal arrest sequences (4). With these common features in mind, we set out conditions of our search for hitherto unidentified arrest peptides of this class in the genomic databases of 449 bacterial species (listed in Supplementary Table S1). We looked for ORFs that meet the following criteria: (i) An ORF of unknown function, which codes for a small (80–200 amino acid residues) protein with no assigned function, and resides in the immediate upstream region of the *sec* or *yidC* gene; (ii) An ORF with a secretion signal sequence or a transmembrane sequence at the N-terminal region, which makes its polypeptide-product a substrate of the *sec* or the *yidC* localization pathway specified by the immediate downstream gene that could be the target of regulation; (iii) An ORF having the C-terminal amino acid sequence mutually conserved among its apparent homologs in bacterial species; (iv) An ORF with the distance between the C-terminal end of the localization sequence and the C-terminal end of the putative arrest sequence >40 amino acid residues to ensure the exposure of the localization signal outside of the peptide exit tunnel of the presumed translation-arrested state of the nascent chain-ribosome complex (Figure 1A).

We first made a list of target bacterial species comprising 449 species. After identification of the *sec* or *yidC* genes by keyword-based search, we manually examined whether the *secY*, *secE*, *secG*, *secA*, *secDF* and *yidC* genes had an upstream ORF. Subsequently we examined whether the ORFs had a topogenic sequences such as an export signal sequence and transmembrane sequence (see Materials and Methods). By comparing protein sequences of the candidate upstream ORFs manually or using the protein BLAST search, we extracted three homology groups of the ORFs (Supplemental Table S7). The first group (referred to as *apcA* for arrest peptide encoded upstream of *yidC* of actinobacteria) was found upstream of the genes for a homolog (presumably the secondary) of YidC (YidC2) in the genomic sequences of a subset of actinobacteria (Figure 1B). This group of ORFs encodes a protein with one or two transmembrane (TM) segment(s) at the N-terminus and a mutually-conserved sequence at the C-terminus (Figure 1C). The membrane topology prediction by the TMHMM program (33) suggests that, regardless of the number of the TM segment, the ApcA proteins have a short extracytoplasmic region and the C-terminal region in the cytosol, a typical structural feature shared among substrates of the YidC insertases (21).

The second and the third groups (referred to as *apdA* for arrest peptide encoded upstream of *secDF* of actinobacteria, and *apdP* for arrest peptide encoded upstream of *secDF* of α-proteobacteria, respectively) were found upstream of the genes for homologs (presumably the secondary) of SecDF (SecDF2) in the genomic sequences of subsets of actinobacteria and α-proteobacteria, respectively (Figure 1B). They encode proteins with an N-terminal hydrophobic sequence and the C-terminal mutually-conserved sequence (Figure 1D and E). The ApdA homologs typically have a putative cleavable signal sequence at the N-terminus. By contrast, the ApdP homologs typically have a putative N-terminal signal-anchor sequence, a TM segment with the Nin-Cout orientation without a signal peptidase cleavage site, although some of them were predicted to have a cleavable signal sequence. It is known that both of these proteins can be handled by the SecYEG-SecDF membrane-embedded complex in conjunction with the SecA ATPase.

### Translation arrest *in vitro*

**Figure 2.**
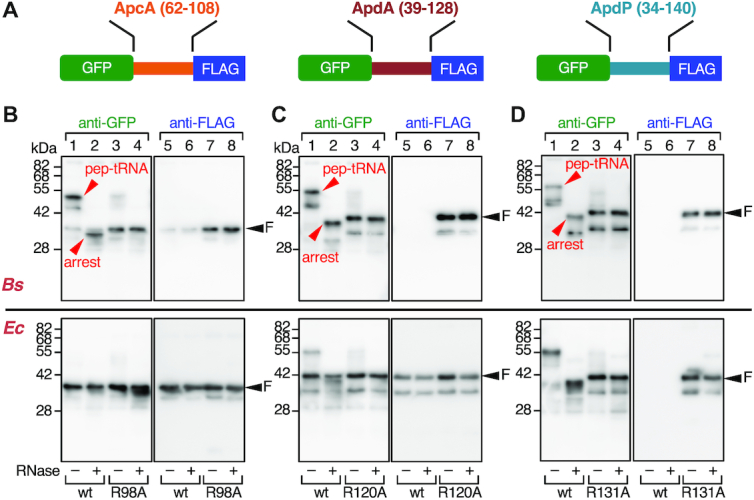
ApcA, ApdA and ApdP arrest translation *in vitro*. (**A**) Schematic representations of GFP-fusion derivatives of ApcA, ApdA and ApdP. The hydrophilic domains of ApcA (residues 62–108), ApdA (residues 39–128) and ApdP (residues 34–140) were sandwiched between the N-terminal GFP and the C-terminal FLAG-tag. (**B**–**D**) Immunoblotting of *in vitro* translation products of the ApcA (B), ApdA (C) and ApdP (D) derivatives. *In vitro* translation using *Bs* hybrid PURE system (upper) or the original (*Ec*) PURE system (lower) was directed by the *gfp–apcA-flag*, *gfp-apdA-flag* and *gfp-apdP-flag* templates with or without the arginine mutations indicated at the bottom. Reaction products were analyzed by SDS-PAGE using the neutral pH (WIDE-RANGE) gel system, followed by immunoblotting with anti-GFP (left) and anti-FLAG (right). To remove the tRNA moiety from polypeptidyl-tRNA, we treated portions of samples with RNaseA before electrophoresis (lanes with even-numbers). ‘pep-tRNA’ and ‘arrest’ indicate peptidyl-tRNA and arrested peptide moieties, respectively, and ‘F’ indicates full-length polypeptide.

We chose *Rhodococcus erythropolis* ApcA (108 residues), *Amycolatopsis japonica* ApdA (128 residues) and *Sinorhizobium medicae* ApdP (140 residues) as representatives of each class for biochemical characterization (Figure 1B, F). To address whether ApcA, ApdA and ApdP arrest their translation, we used *in vitro* coupled transcription-translation reactions. PURE system contains the purified components for translation derived from *E. coli* (39), whereas its modified version, *Bs* hybrid PURE system, contains the *B. subtilis* ribosomes instead of the *E. coli* ribosome (12). We designed templates for *in vitro* translation in the following ways. We sandwich-fused each of the gene segments for the C-terminal hydrophilic domains of ApcA (residues 62–108), ApdA (residues 39–128) and ApdP (residues 34–140) between the GFP-coding sequence (N-terminal side) and the FLAG-tag (C-terminal side) (Figure 2A). Translation-arrested products can be distinguished from translation-terminated products by the presence of a tRNA covalently attached to the growing end of the nascent polypeptide, which retards electrophoretic mobility upon SDS-PAGE separation at neutral pH (24). RNase treatment before electrophoresis causes downward band shift of the arrested products but not the normal translation products. For neutral pH electrophoresis, we used the WIDE RANGE gel system (42).

*In vitro* translation of GFP-ApcA-FLAG using *Bs* hybrid PURE system resulted in the production of a major product detected by anti-GFP immunoblotting at the position slightly lower than the 55 kDa size marker (Figure 2B, lane 1; arrowhead with ‘pep-tRNA’). The mobility of this product exhibited a marked downshift to the position between the 28 and 42 kDa markers when the SDS-PAGE sample had been pretreated with RNaseA (Figure 2B, upper panel, lane 2; arrowhead with ‘arrest’). Consistent with this product representing an outcome of translation arrest, the bands, both with or without RNase treatment, were undetectable by anti-FLAG immunoblotting; the product lacks the C-terminal FLAG-tag (Figure 2B, upper panel, lanes 5 and 6). We also observed a minor band between the 28 and 42 kDa markers regardless of the RNase-pretreatment, which was detected by both anti-GFP and anti-FLAG (Figure 2B, upper panels, lanes 1, 2, 5 and 6; arrowhead with ‘F’). Probably, the minor product was the full-length GFP-ApcA-FLAG protein, which was expected to be produced by spontaneous arrest release during the reaction. These results show that the ApcA sequence in the fusion protein potently arrests translation elongation by the *B. subtilis* ribosomes. The migration position of the polypeptide fragment that lacks the tRNA (Figure 2B, upper panel, lane 2) is slightly lower than that of full-length species, suggesting that the translation arrest occurs within the ApcA-coding sequence near the stop codon. We show the importance of the conserved ApcA sequence in the C-terminal region by replacing the conserved arginine residue (Arg98) with alanine. The R98A mutation largely abolished the production of the RNase-sensitive arrest species, with concomitant elevation in the production of the full-length species (Figure 2B, upper panels, lanes 3, 4, 7 and 8). Strikingly, *in vitro* translation of GFP-ApcA-FLAG by the *E. coli* PURE system (*Ec* PURE system) resulted in the almost exclusive production of the full-length species, regardless of the presence or absence of the R98A mutation (Figure 2B, lower panels, lanes 1–8). Thus, ApcA stalls the *B. subtilis* ribosome but not the *E. coli* ribosome.

*In vitro* translation of GFP-ApdA-FLAG and GFP-ApdP-FLAG using *Bs* hybrid PURE system also resulted in the production of RNase-sensitive arrest species (Figure 2C and D, upper panels, lanes 1, 2, 5 and 6) as the major products. We note that sometimes two arrest bands (typically, Figure 2C, upper panel lane 1) were produced from the same template. The lower band may have been due to incomplete unfolding of the GFP moiety, which could cause faster electrophoretic migration than completely unfolded and extended species, or, alternatively, internal translation initiation. Remarkably, neither anti-GFP nor anti-FLAG detected any full-length products unless Arg120 of ApdA or Arg131 of ApdP was mutated to alanine (Figure 2C and D, upper panels, lanes 1–8). These results indicate that ApdA and ApdP strongly arrest translation by *B. subtilis* ribosome such that the full-length products were produced only negligibly. *In vitro* translation of GFP-ApdA-FLAG using the original *Ec* PURE system resulted in the accumulation of the full-length species as the major product and arrested species as a minor product (Figure 2C, lower panels, lanes 1, 2, 5 and 6). The level of the arrested species further decreased when the R120A mutation was introduced (Figure 2C, lower panels, lanes 3, 4, 7 and 8). By contrast, *in vitro* translation of GFP-ApdP-FLAG using the *Ec* PURE system produced the arrest species almost exclusively. These results reveal that ApdA preferentially acts against the *B. subtilis* ribosomes over the *E. coli* ribosome, whereas ApdP is equally effective in stalling the *B. subtilis* and the *E. coli* ribosomes. Taken together, we conclude that these three proteins, in the ribosome-tethered nascent states, are capable of arresting their translation elongation.

### *In vivo* translation of the new arrest peptides

**Figure 3.**
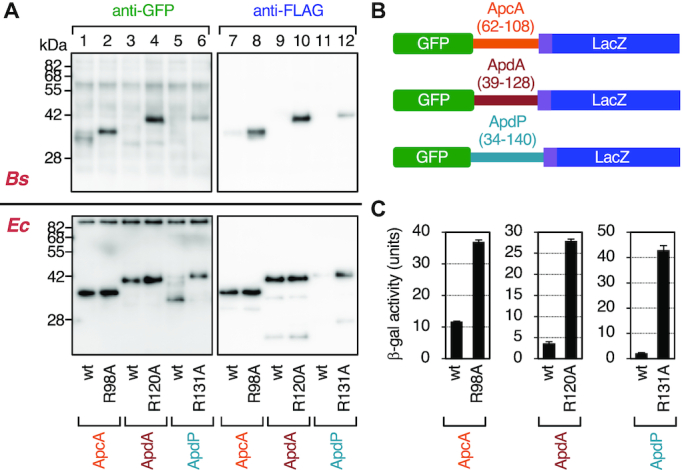
*In vivo* translation arrest of the ApcA, ApdA and ApdP derivatives. (**A**) Immunoblotting of *in vivo* translation products of the ApcA, ApdA and ApdP derivatives. GFP-ApcA-FLAG (lanes 1, 2, 7, 8), GFP-ApdA-FLAG (lanes 3, 4, 9, 10) and GFP-ApdP-FLAG (lanes 5, 6, 11, 12) with or without the arginine mutations, indicated at the bottom, were expressed in *B. subtilis* (upper) or *E. coli* (lower). Proteins in the cell extracts were analyzed by immunoblotting using anti-GFP (left) and anti-FLAG (right). The tRNA moiety of the arrest product had been removed during the course of sample preparation. (**B**) Schematic representations of the *gfp-apcA-lacZ*, *gfp-apdA-lacZ* and *gfp-apdP-lacZ* reporters. (**C**) β-galactosidase assay of the wildtype and arrest defective mutant derivatives of the *gfp-apcA-lacZ*, *gfp-apdA-lacZ* and *gfp-apdP-lacZ* reporters. Cell cultures of *B. subtilis* strains expressing wildtype or the arginine mutant derivatives of the above *lacZ* reporters were harvested and subjected to β-galactosidase assay (mean ± SD, *n* = 3). The mutations introduced are indicated at the bottom.

We next tested whether ApcA, ApdA and ApdP arrest translation *in vivo*. We expressed GFP-ApcA-FLAG, GFP-ApdA-FLAG and GFP-ApdP-FLAG in *B. subtilis* and *E. coli* cells. Their translation products were examined by western blotting following SDS-PAGE separation. Note that we intended to detect the *in vivo* proteins after removing any tRNA moiety that could be present in their arrested/nascent states. Being consistent with the *in vitro* translation assay, cell lysates of *B. subtilis* expressing any one of the tripartite fusion proteins did not contain detectable levels of the full-length products, which were reactive with anti-FLAG antibody (Figure 3A upper panels, lanes 7, 9 and 11). This failure was not due to insufficient expression of these proteins, because their arginine mutant forms were detected as the full-length species (Figure 3A, upper panels, lanes 8, 10 and 12). Anti-GFP immunoblotting detected a GFP-ApcA-FLAG-related band that migrated slightly faster than the full-length species observed for the R98A mutant form (Figure 3A, upper panel, lanes 1 and 2). This band, undetectable by anti-FLAG (lane 7), most likely represented the elongation-arrested GFP-ApcA' product. However, we could not detect corresponding arrest bands of GFP-ApdA-FLAG or GFP-ApdP-FLAG *in vivo* (Figure 3A, upper panel, lanes 3 and 5), possibly due to proteolytic degradation of the arrested species when they are synthesized in *B. subtilis*. Nevertheless, the arginine mutated variants of these proteins accumulated as the full-length species in the cell and were detected immunologically. These results are consistent with the results of the *in vitro* translation experiments, which detected the full-length species only when the conserved arginine residue was mutated.

When expressed in *E. coli*, the full-length species of GFP-ApcA-FLAG and GFP-ApdA-FLAG accumulated in the cell regardless of the presence or absence of the mutation (Figure 3A, lower panel, lanes 1–4, 7–10). By contrast, the full-length species of GFP-ApdP-FLAG did not accumulate significantly in *E. coli* cells (Figure 3A, lower panel, lanes 5 and 11) unless it had the R131A mutation (lanes 6 and 12). Consistent with the *in vitro* observation that GFP-ApdP-FLAG undergoes elongation arrest when translated either by the *E. coli* or the *B. subtilis* ribosomes, we were able to detect a prominent GFP-ApdP-FLAG-related product that migrated faster than the full-length species and was reactive with anti-GFP (Figure 3A, lower panel, lanes 5), but not with anti-FLAG (lane 11) in *E. coli* cells. The migration position of this presumed arrest product was lower than that expected for the arrest band with a denatured GFP moiety. The reason for this electrophoretic anomaly is unknown. Perhaps, proteolytic cleavage of the arrested GFP-ApdP' nascent chain could account for the observation. The results obtained from these *in vivo* analyses are consistent with the conclusion reached by the *in vitro* analysis that ApcA, ApdA and ApdP are all competent in arresting their translation elongation by the *B. subtilis* ribosomes, but only ApdP impacts the *E. coli* ribosomes.

As an independent assessment of elongation arrest *in vivo*, we constructed fusion proteins featuring the LacZ sequence as a reporter of translation continuation beyond the putative arrest sequence. We fused the *lacZ* sequence in-frame after the 3′ end of the FLAG-tag coding sequence of the constructs used above. The resulting in-frame fusion genes encoding GFP-ApcA(62–108)-FLAG-LacZ, GFP-ApdA(39–128)-FLAG-LacZ and GFP-ApdP(34–140)-FLAG-LacZ (hereafter referred to as GFP-ApcA-LacZ, GFP-ApdA-LacZ and GFP-ApdP-LacZ, respectively; Figure 3B) were introduced into the chromosomal DNA of *B. subtilis*. Elongation arrest within the ApcA, ApdA or ApdP coding region will block translation continuation to the downstream *lacZ*, resulting in low β-galactosidase activity. As controls, we used the arginine to alanine mutations that were shown to compromise the elongation arrest. As shown in Figure 3C, the β-galactosidase activities of GFP-ApcA-LacZ, GFP-ApdA-LacZ and GFP-ApdP-LacZ, expressed in *B. subtilis* cells, were lower than those of their R98A, R120A and R131A mutant forms by 3.2-, 7.6- and 19.5-fold, respectively. These results, taken together, show that the amino acid sequences of ApcA, ApdA and ApdP contain determinants of elongation arrest that takes place in certain bacterial species.

### Frameshift and synonymous mutation analyses of the importance of the amino acid sequences in the elongation arrest

To examine the importance of the amino acid sequence of the C-terminal hydrophilic domains of ApcA, ApdA and ApdP in the elongation arrest, we introduced internal frameshift mutations, which drastically alter the amino acid sequences but only minimally the mRNA sequences. To evaluate the mutational effects quantitatively, we used the in-frame LacZ fusion proteins described above. We introduced internal frameshifts into a defined segment of the ApcA, the ApdA, or the ApdP-coding region by deleting the first nucleotide of the target segment and inserting a nucleotide after the end of it. For instance, GFP-ApcA_FS62–108-LacZ was constructed by deleting the first nucleotide of the 62nd codon and inserting a nucleotide after the 108th codon of *apcA* (the codon numbers correspond to those in native *apcA*). The mutant construct has the ApcA 62–108 amino acid sequence replaced with a totally unrelated sequence. In cases where a frameshift was expected to create an in-frame stop codon in the target segment, we introduced a synonymous mutation into the problematic codon beforehand to avoid the stop codon appearance.

**Figure 4.**
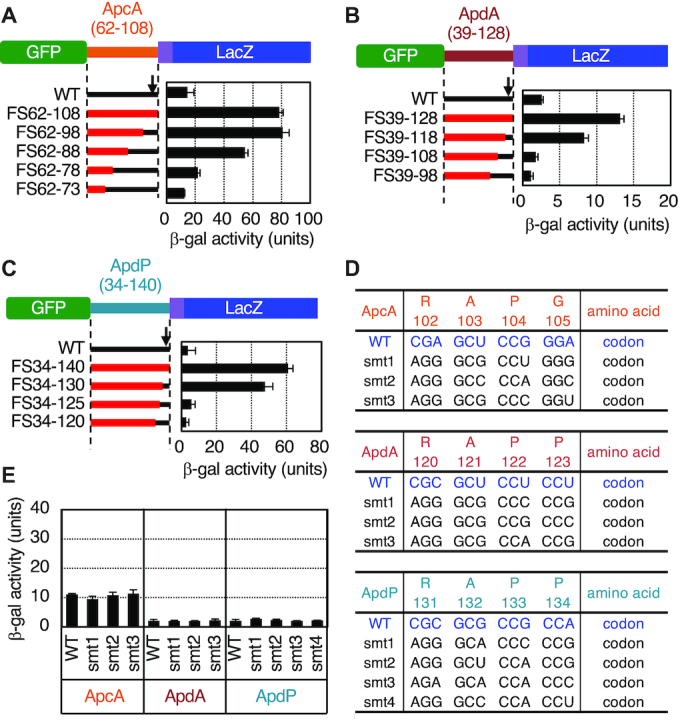
Internal frameshift mutations in the C-terminal regions of ApcA, ApdA and ApdP alleviate the elongation arrest. (**A**–**C**) Effects of frameshift mutations in ApcA (A), ApdA (B) and ApdP (C) on the efficiencies of the elongation arrest. Internal frameshift mutations were introduced into the ApcA, ApdA and ApdP coding regions of the *gfp-apcA-lacZ*, *gfp-apdA-lacZ* and *gfp-apdP-lacZ* reporters. Cell cultures of *B. subtilis* strains expressing wildtype or mutant derivatives of the above *lacZ* reporters were harvested and subjected to β-galactosidase assay. The frameshifted segments are indicated in red with numbers indicating the first and the last frameshifted codons. The right panels show β-galactosidase activities (mean ± SD, *n* = 3) of *B. subtilis* cells expressing the reporter genes indicated on the left. The black arrows indicate the positions of the RAP[G/P] sequences shown in (D). (**D**) Amino acid and nucleotide sequences of the synonymous mutant derivatives of ApcA, ApdA and ApdP. ‘smt’ indicates a synonymous mutant. The amino acid numbering is indicated below the sequences. (**E**) β-galactosidase activities (mean ± SD, *n* = 3) of *B. subtilis* cells expressing the *gfp-apcA-lacZ*, *gfp-apdA-lacZ* and *gfp-apdP-lacZ* fusion proteins with the synonymous mutations indicated in (D).

*B. subtilis* cells expressing the wildtype GFP-ApcA-LacZ exhibited a low level of β-galactosidase activity (14.6 units; Figure 4A). Introduction of the internal frameshift mutation to alter the amino acid sequence of the whole C-terminal cytosolic region of ApcA (residues 62–108) markedly elevated the β-galactosidase activity (78.4 units; Figure 4A). These results support the notion that the efficient elongation arrest requires the amino acid sequence of the C-terminal cytoplasmic region of ApcA. We then varied the C-terminal ends of frameshifts. As the endpoints were shifted toward the N-terminus, the β-galactosidase activities gradually decreased until the position 73; cells expressing GFP-ApcA_FS62–73-LacZ proved to have a level of β-galactosidase activity (12.3 units, Figure 4A) that was as low as that of the construct without the mutation. Thus, the ApcA region corresponding to the residues 74–108 is sufficient for the full execution of elongation arrest.

We carried out similar analyses for ApdA and ApdP using *B. subtilis* cells expressing the wildtype and frameshifted derivatives of GFP-ApdA-LacZ and GFP-ApdP-LacZ (Figure 4B and C). β-galactosidase activities of cells expressing ApdA and ApdP were 2.6 and 3.6 units, respectively (Figure 4B and C), which were increased to 13.2 units and 60.8 units, respectively, by the frameshift mutations affecting the whole ApdA and ApdP hydrophilic sequences (ApdA_FS39–128 and ApdP_FS34–140). While GFP-ApdA_FS39–118-LacZ still had a high level of β-galactosidase activity, GFP-AdpA_FS39–108-LacZ and GFP-AdpA_FS39–98-LacZ had the ‘wildtype’ levels of β-galactosidase activity (Figure 4B). These results suggest that the C-terminal 20 residue segment of ApdA (residues 108–128) is required and sufficient for the elongation arrest. Cells expressing GFP-ApdP_FS34–130-LacZ and GFP-ApdP_FS34–125-LacZ exhibited higher levels of β-galactosidase activity than those expressing the corresponding wildtype construct, whereas GFP-ApdP_FS34–125-LacZ and GFP-ApdP_FS34–120-LacZ exhibited nearly wildtype levels of the activity (Figure 4C). These results narrow down the arrest sequence of ApdP to the C-terminal 15–20 amino acid residues.

The results of the frameshift experiments suggest that the amino acid sequences of the new arrest peptides, but not the features of their mRNAs, are crucial for the elongation arrest that takes place in their translation. To complement this conclusion, we also tested the impacts of synonymous mutations introduced into selected four consecutive codons of the arrest peptides (R^102^APG^105^ of ApcA, R^120^APP^123^ of ApdA, and R^131^APP^134^ of ApdP) in the *lacZ* reporter context (Figure 4D). We chose these segments because they are highly conserved among their homologs, and substitution of each single amino acid within these regions alleviated the elongation arrest (see below). The nucleotide alterations of the four codons in the reporter genes without amino acid changes (Figure 4D) did not significantly elevate the β-galactosidase activity (Figure 4E), supporting our conclusion that the amino acid sequences of the nascent polypeptides, rather than nucleotide sequences of mRNA, are primarily responsible for the elongation arrest.

### Determination of ribosomal stalling sites by toeprint experiments

**Figure 5.**
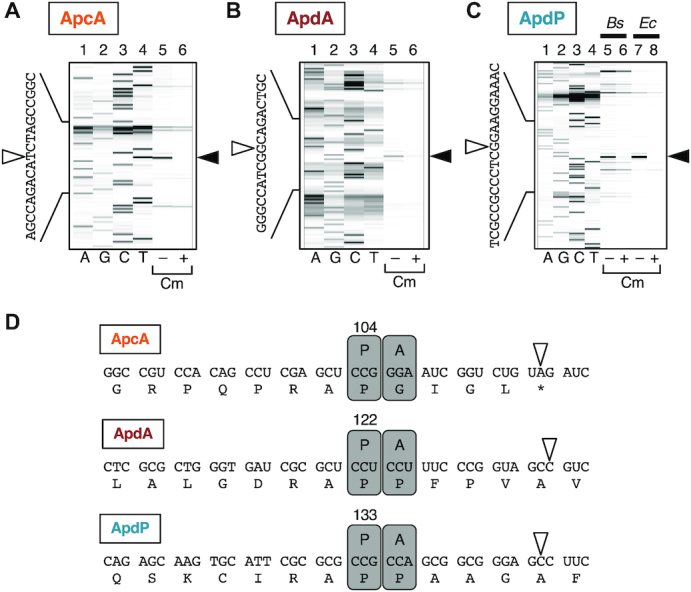
Determination of the ribosomal stalling sites on the *apcA*, *apdA* and *apdP* mRNA by toeprinting. (**A–C**) Toeprint analysis of *apcA* (A), *apdA* (B) and *apdP* (C). Coupled *in vitro* transcription-translation was directed with the *gfp-apcA* (A), *gfp-apdA* (B) and *gfp-apdP* (C) templates using *Bs* PURE system (A-C; lanes 5, 6) and the original (*Ec*) PURE system (C; lanes 7, 8) in the presence or the absence of 0.1 mg/ml chloramphenicol (Cm) as indicated at the bottom. Translation complexes were then subjected to reverse transcription using a fluorescent primer. The reverse transcripts were separated by the capillary electrophoresis and visualized by HiTRACE online software. Lanes A, G, C and T indicate respective dideoxy sequencing products. The black and white arrowheads represent the major toeprint signals and their last nucleotides, respectively. (**D**) Cartoon representation of the positions of the ribosomes stalled on the *apcA*, *apdA* and *apdP* mRNAs. Boxes ‘P’ and ‘A’ represent the residence of the P-site and A-site of the ribosome, respectively, on the mRNAs. The numbers above the boxes ‘P’ indicate the P-site codons. White arrowheads indicate the last nucleotides of the reverse transcription products observed upon toeprint assays.

To determine the ribosomal stalling sites on the *apcA*, *apdA* and *apdP* mRNAs, we undertook toeprint experiments. Reaction mixtures of *in vitro* translation of the *gfp*-fused derivatives of *apcA*, *apdA* and *apdP* using *Bs* hybrid PURE system as well as that of *apdP* using *the Ec* PURE system were subjected to reverse transcription using primers complementary to the 3′ regions of mRNAs. Toeprint signals were generated by interruption of the primer extension reaction by the stalled ribosomes on the template mRNA (Figure 5A and B, lane 5, Figure 5C, lanes 5 and 7). Among several bands observed for each template, the prominent signals, highlighted by filled arrowheads, were specific, as they were not appreciably observed in the negative control samples, in which a translation inhibitor chloramphenicol (Cm) had been added during *in vitro* translation (Figure 5A and B, lane 6, Figure 5C, lanes 6 and 8). We determined the points of reverse transcription abortion from the sizes of the translation-specific toeprint products (Figure 5A–C, black arrowheads), which were calibrated by dideoxy sequencing patterns obtained by the same primer as used for toeprinting (Figure 5A–C, lanes 1–4, Figure 5A–D, white arrowheads). As a stalled ribosome stops reverse transcription at the position 15–17 nucleotides downstream of the first nucleotide of the P-site codon (43), we assign the positions of the ribosomal stall to codon 104 for ApcA, codon 122 for ApdA, and codon 133 for ApdP, each of which occupies the P-site (Figure 5D).

### Arrest peptides block peptidyl transfer to the next aminoacyl-tRNA or the A-site accommodation of the latter

**Figure 6.**
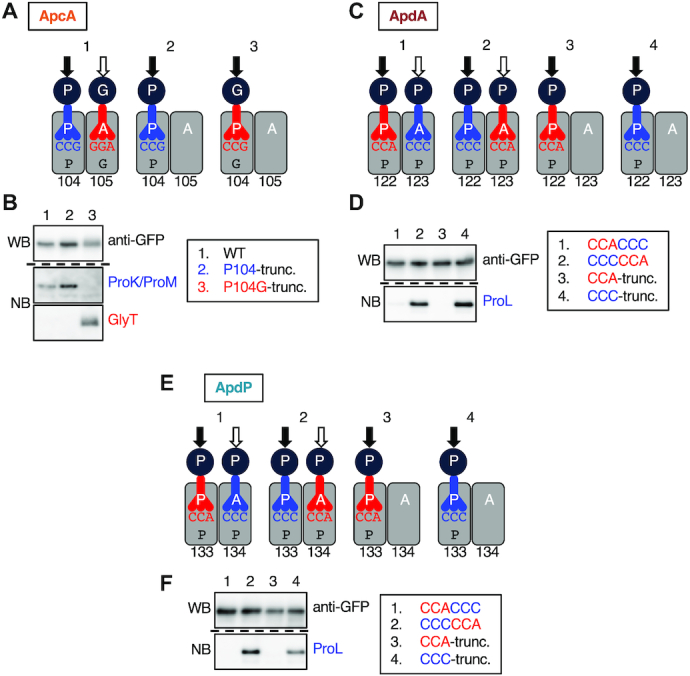
Nascent chains impair the peptidyl transfer. (**A**) Cartoon representations of translation intermediates of the *apcA* (1), and its derivatives with the mRNA truncation after the 104th codon (*apcA-P104-trunc*; 2), and its P104G version (*apcA-P104G-trunc*; 3). (**B**) Western and northern blotting of ApcA’-tRNAs. The *gfp-apcA* fusion gene (lane 1) was subjected to *in vitro* translation reaction using the *Bs* hybrid PURE System. As controls, we also used *gfp-apcA* mRNA truncated after the 104th codon (lane 2) and its P104G version (lane 3) as a template. The products were separated by 11% WIDE RANGE gel, and the regions where ApcA’-tRNA molecules were expected to migrate were analyzed by anti-GFP immunoblotting (Upper) and northern blotting using specific probes for tRNA^Pro^ (ProK and ProM probes; middle) and tRNA^Gly^ (GlyT probe; lower). (**C**) Cartoon representations of translation intermediates of the *apdA* with CCA-CCC sequence (1) and CCC-CCA sequence (2) for the codons 122 and 123, and their derivatives with the truncation after the 122nd codon (3, 4). (**D**) Western and Northern blotting of ApdA’-tRNAs. The *gfp-apdA* fusion genes with CCA-CCC (lane 1) and CCC-CCA (lane 2) sequences, and their truncated variants after the 122nd codon (lanes 3, 4) were subjected to *in vitro* translation reaction using the *Bs* hybrid PURE System. The translation products were analyzed by anti-GFP immunoblotting (Upper) and Northern blotting using a specific probe for the tRNA^Pro^ that decodes the CCC codon (ProL probe; Lower). **(E)** Cartoon representations of translation intermediates of the *apdP* with CCA-CCC sequence (1) and CCC-CCA sequence (2) for the codons 133 and 134, and their derivatives with the truncation after the 133rd codon (3, 4). (**F**) Western and Northern blotting of ApdP’-tRNAs. The *gfp-apdP* fusion genes with CCA-CCC (lane 1) and CCC-CCA (lane 2) sequences, and their truncated variants after the 133rd codon (lanes 3, 4) were subjected to *in vitro* translation reaction using the *Bs* hybrid PURE System. The translation products were analyzed by anti-GFP immunoblotting (Upper) and Northern blotting using a specific probe for the tRNA^Pro^ that decodes the CCC codon (ProL probe; Lower). Black and white arrows in (A), (C), (E) indicate positions of the nascent chain before and after the peptidyl transfer, respectively.

Our toeprint analysis indicates that the Pro104 and Gly105 codons of *apcA* reside at the P-site and A-site of the stalled ribosome, respectively. If the elongation arrest of ApcA occurs by a peptidyl transfer dysfunction, the arrest product should carry tRNA^Pro^. By contrast, if the arrest is due to a defect in the post-peptidyl-transfer step, the arrest product should carry tRNA^Gly^ (Figure 6A; cartoon 1). We determined the identity of the tRNA moiety on the arrested GFP-ApcA peptidyl-tRNA, produced by *in vitro* translation using *Bs* hybrid PURE system followed by Northern blotting using specific DNA probes that can distinguish between tRNA^Pro^ and tRNA^Gly^. The specificity of the probes was confirmed by using truncated DNA templates to produce defined peptidyl-tRNAs upon translation. The truncation after codon 104 (P104-trunc) produces peptidyl-prolyl-tRNA, and its Pro104Gly derivative (P104G-trunc) produces peptidyl-glycyl-tRNA (Figure 6A, cartoon 2 and 3). *In vitro* translation of P104-trunc and P104G-trunc confirmed the production of GFP-ApcA’-tRNA^Pro^ and GFP-ApcA’-tRNA^Gly^, which were detected by anti-GFP Western blotting (Figure 6B, top panel, lanes 2 an 3) and specifically with respective probes upon northern blotting (Figure 6B, middle and bottom panels, lanes 2 and 3). The translation product of ApcA was highlighted with anti-GFP (Figure 6B, top panel, lane 1) as well as with the tRNA^Pro^ probe (Figure 6B, middle panel, lane 1) but not the tRNA^Gly^ probe (Figure 6B, bottom panel, lane 1). Thus, elongation of ApcA is arrested in a state in which peptidyl-prolyl-tRNA resides at the P-site of the ribosome, indicating that peptidyl transfer beyond the Pro104 residue is blocked even if the following glycyl-tRNA is already in the A-site, or alternatively, the A-site cannot accommodate glycyl-tRNA.

We carried out similar experiments for ApdA and ApdP. Our toeprint analysis revealed that both A-site and P-site codons of ApdA and ApdP specify proline. Thus, we mutated the P-site and A-site codons such that they are decoded by different isoacceptors of tRNA^Pro^. We constructed two GFP-ApdA derivatives, one of which with the CCA-CCC codon pair for the 122nd and 123rd codons, and the other with a CCC–CCA codon pair (Figure 6C; cartoon 1, 2). We also constructed their truncated templates lacking codon 123 and its downstream to verify the specificity of DNA probes (Figure 6C; cartoon 3, 4). *In vitro* translation of both the CCA–CCC and the CCC–CCA versions of GFP-ApdA produced peptidyl-tRNA products, which were highlighted with anti-GFP (Figure 6D, upper panel, lanes 1 and 2). However, only the latter was highlighted with the ProL probe (Figure 6D, lower panel, lanes 1 and 2), whose specificity toward the *proL*-encoded and CCC-decoding tRNA^Pro^ was confirmed by the control samples using the truncated templates (Figure 6D, lower panel, lanes 3 and 4). Essentially the same results were obtained for ApdP (Figure 6E and F); the arrest product of the CCC–CCA version was detected by the ProL probe. These results indicate that the arrested nascent chain-ribosome complexes of ApcA, ApdA and ApdP all carry their peptidyl-tRNA products at the P-site of the ribosome, suggesting a common biochemical basis of their elongation arrest.

### Determination of crucial residues for the elongation arrest

We carried out alanine-scanning mutagenesis to identify amino acid residues crucial for the elongation arrest. The frameshift experiments narrowed down segments in the arrest peptides that are essential for the arrest (Figure 4). To assess the contribution of each amino acid in the segments, we introduced individual alanine substitutions to residues 74–104 of ApcA, residues 109–122 of ApdA and residues 126–133 of ApdP. The most C-terminal target residues were selected to coincide with those specified by the P-site codons determined by toeprinting (Figure 5). We replaced each residue of the segments in the GFP-ApcA-LacZ, GFP-ApdA-LacZ, GFP-ApdP-LacZ reporter constructs with alanine or serine (when the target was alanine).

**Figure 7.**
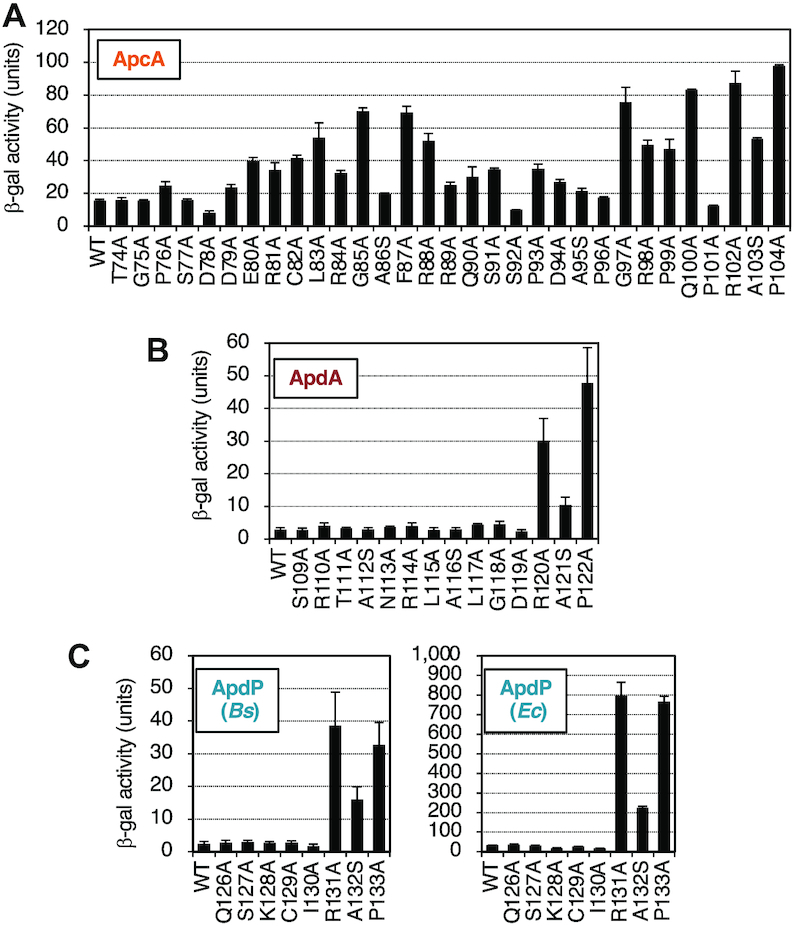
Determination of crucial residues of ApcA, ApdA and ApdP by alanine-scanning mutagenesis. (**A**–**C**) Effects of alanine substitutions within the ApcA (A), ApdA (B), and ApdP (C) regions on the β-galactosidase activities of strains expressing the arrest reporters, *gfp-apcA-lacZ*, *gfp-apdA-lacZ*, *gfp-apdP-lacZ*, and their derivatives. *B. subtilis* strains expressing the *apcA* (A), *apdA* (B) and *apdP* (C; left) arrest reporters, and *E. coli* strains expressing the *apdP* arrest reporters (C; right) were cultured, and β-galactosidase activities (mean ± SD, *n* = 3) were determined. The mutations introduced are indicated at the bottom.

Mutations of ApcA revealed that more than ten of them elevated the β-galactosidase activity by three-fold or more, indicative of arrest deficiency (Figure 7A). Notably, the seven arrest-site-proximal mutations, expect at Pro101, gave markedly increased β-galactosidase activity, indicative of rather severe defects in the arrest. The Pro101 result was somewhat unexpected in view of the translation-intractable nature of proline (44,45). Mutations of several residues distal to the arrest site (such as L83A, G85A, F87A and R88A) also increased the β-galactosidase activity, indicating that the elongation arrest of ApcA requires both the arrest site-proximal and arrest site-distal residues. We note that the D78A and S92A mutations gave β-galactosidase activity that was lower than that of the ‘wildtype’ construct (Figure 7A), raising a possibility that these mutations enhance the arrest proficiency of ApcA. In summary, alanine-scanning mutagenesis of ApcA highlighted the importance of both the arrest site-proximal and arrest site-distal residues of ApcA. The elongation arrest of ApcA depends on the nascent chain-ribosome interactions near the PTC as well as in the polypeptide exit tunnel of the ribosome.

In the cases of ApdA and ApdP, by contrast, only mutations at the arrest point-proximal three residues of each arrest peptide proved to affect the elongation arrest. They are the ApdA residues Arg120, Ala121 and Pro122 (Figure 7B) and the ApdP residues Arg131, Ala132 and Pro133 (Figure 7C, left panel). However, it is unlikely that these three residues are the sufficient determinants of the arrest. The frameshift analysis of GFP-ApdA-LacZ has shown that FS_39–118 still elevated the reporter activity, whereas FS_39–108 did not. Thus, it is suggested that the interval of residues 109 to 118 has some element required for the elongation arrest in ApdA (Figure 4B). Similarly, frameshift mutagenesis of GFP-ApdP-LacZ showed that the FS_34–130 mutation but not FS_34–125 increased the β-galactosidase activity (Figure 4C). Thus, the interval of residues 126–130 of ApdP might also play a role in the elongation arrest of ApdP. Essentially the same alanine scanning results were obtained when we expressed the ApdP *lacZ* reporters in *E. coli* (Figure 7C, right panel). In summary, ApdA and ApdP appear to have shorter arrest sequences near the arrest point. Notably, the three amino acid residues near the PTC are strictly required, such that their single amino acid substitutions abolish the elongation arrest.

### The requirement of A-site codon for the elongation arrest

**Figure 8.**
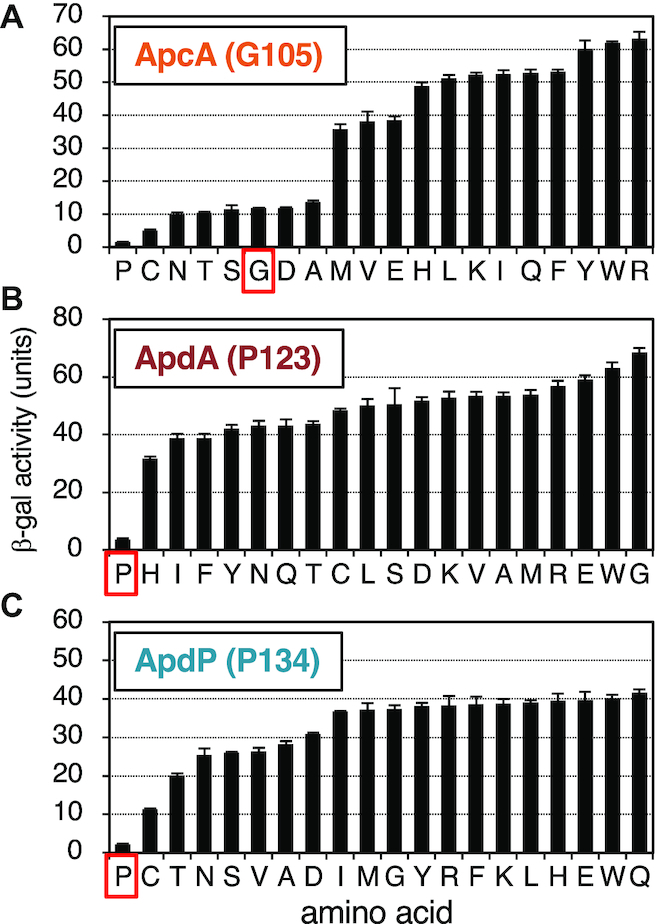
Impact of the amino acids encoded by the A-site codons for the efficiency of the elongation arrest of ApcA, ApdA and ApdP. (**A**–**C**) Amino acid residues encoded by the A-site codons of ApcA (Gly105), ApdA (Pro123) and ApdP (Pro134) in the *gfp-apcA-lacZ* (A), *gfp-apdA-lacZ* (B) and *gfp-apdP-lacZ* (C) reporters were mutated to all other possible amino acids indicated at the bottom. The wildtype and mutant derivatives of the *lacZ* reporter genes were expressed in *B. subtilis*, and β-galactosidase activities (mean ± SD, *n* = 3) were determined. Red squares indicate wildtype strains.

The results so far presented show that the nascent peptidyl-tRNAs of the arrest peptides at the P-site somehow impair the ribosomal function of chain elongation. It remains to be elucidated whether the A-site aminoacyl-tRNAs have any roles in the elongation arrest. To address this point, we systematically mutated the A-site-specified amino acid of ApcA (Gly105), ApdA (Pro123) and ApdP (Pro134) to the other 19 amino acids in the *gfp-apcA-lacZ*, *gfp-apdA-lacZ* and *gfp-apdP-lacZ* gene contexts. We then measured the β-galactosidase activity of *B. subtilis* expressing the wildtype and the above mutant derivatives of the *lacZ* reporters. Eleven mutants of ApcA (G105M, G105V, G105E, G105H, G105L, G105K, G105I, G105Q, G105F, G105Y, G105W, G105R) had elevated levels of β-galactosidase activity, indicative of arrest impairment, whereas five mutants (G105N, G105T, G105S, G105D, G105A) had the wildtype levels (Figure 8A). Interestingly, two mutants (G105P and G105C) exhibited lower than wildtype levels of β-galactosidase activity, suggesting that these mutations, G105P in particular, enhanced the elongation arrest of ApcA. In AdpA and ApdP, by contrast, all the mutations at Pro123 (AdpA) and Pro134 (ApdP) led to the elevation of β-galactosidase activity, indicating that the proline codon is uniquely required at the A-site for the execution of elongation arrest (Figure 8B and C).

These mutation studies suggest that the identity of the A-site codon is important for the elongation arrest of ApcA, ApdA and ApdP. In particular, the A-site codon of the ApdA and ApdP nascent chain-ribosome complexes must specify proline. Interestingly, the wildtype glycyl-tRNA was not absolutely required at the A-site of the ApcA nascent chain-ribosome complex, which can be either prolyl-, cysteinyl-, asparaginyl-, threonyl-, seryl-, aspartyl- or alanyl-tRNA. Remarkably, proline works best among all the possible amino acids at residue 105 of ApcA. It is noteworthy that ApdA and ApdP share the same arrest site-proximal sequence, R-A-P-P, which is essential for elongation arrest. Although the arrest site-proximal sequence of ApcA is R-A-P-G, R-A-P-P is the better substitute at this position of this arrest peptide. RAPP could be a part of broader-scope arrest sequences.

## DISCUSSION

Amino acid sequence-based homology search is a powerful bioinformatics approach to detect a family of proteins sharing a common function. However, translation arrest peptides thus far identified are diverse in amino acid sequences despite similar functions of their nascent state to impair translation activity of the ribosome (1–3). The lack of obvious sequence similarity challenges bioinformatic prediction or identification of new arrest peptides. In this study, we took advantage of the monitoring substrates, the molecules of our interest, of having additional characteristic features that should aide bioinformatic analysis (4). Specifically, their chromosomal locations are limited to the N-terminal vicinity of genes for Sec or YidC components, and their N-terminal regions should include cognate topogenic sequences. Although we did not impose any limitation on sequence features of the C-terminal regions, where arrest sequences were expected to reside, we did pick up those having similar C-terminal sequences within the homologous peptides found among related bacterial subspecies. By genetic and biochemical characterization, we confirmed that ApcA, ApdA and ApdP, thus identified bioinformatically, indeed have elongation-arresting properties. Together with the previously identified Sec- or YidC-related arrest peptides, we now have a total of six classes of them encoded upstream of *sec* or *yidC* genes. Although it remains to be studied whether ApcA, ApdA and ApdP indeed feedback-regulate the expression of their downstream genes in their native bacterial strains, they share crucial features with the known monitoring substrates. However, RNA secondary structure prediction programs did not reveal the formation of mRNA hairpin structures that could sequester the SD sequences of the downstream genes. Thus, ApcA, ApdA and ApdP could use regulatory mechanisms that are distinct from those used by the previously reported monitoring substrates. Further experiments are required to elucidate the physiological functions of the new arrest peptides identified in this study.

While ApcA and ApdA selectively arrested the translation elongation by the *B. subtilis* ribosome without affecting the *E. coli* ribosome, ApdP was effective to halt elongation by the ribosomes from both bacterial species. The observed species-specificity of the ApcA and ApdA actions are consistent with the fact that these arrest peptides occur in Gram-positive bacteria, to which *B. subtilis* belongs. Species-specificity was also observed previously in the elongation arrest of *B. subtilis* MifM; it affects the *B. subtilis* ribosome but only weakly the *E. coli* ribosome (12). By contrast, ApdP showed a broader specificity as it affected both the *B. subtilis* and the *E. coli* ribosomes. One factor that might contribute to the species-specificity would be the length of the arrest-essential regions in the nascent peptide. An arrest peptide with a longer arrest sequence undergoes extensive interactions with the ribosomal components at its multiple residues in order to exhibit the elongation-arresting activity. Therefore, such a nascent chain having a long arrest sequence may be more sensitive to the fine structural differences of the ribosomes, with which it interacts to induce conformational changes. Indeed, the species-specific ApcA has the longest arrest sequence, whereas a more universal arrest peptide, ApdP has the shortest arrest sequence among the three arrest peptides examined in this study. The elongation arrest of MifM, another species-specific arrest peptide, depends on extensive interactions with the PTC to the ribosomal surface components or the ribosome (7,31,40), in line with the above discussion.

It is likely that ApcA, ApdA and ApdP induce elongation arrest by their nascent chains' direct interactions with the ribosome. Their arrest-provoking sequences are short enough to be fully accommodated within the exit tunnel of the stalled ribosomes, precluding any involvement of a *trans*-acting cellular factor that interacts with the arrest sequences to execute an efficient arrest. In fact, the elongation arrest occurs efficiently in the PURE system containing a minimal set of non-ribosomal translation factors derived from *E. coli*.

Our toeprint and Northern blot results show that ApcA, ApdA and ApdP elicit ribosome stalling at a single site on each mRNA. The single-site stalling is observed for most of the known regulatory arrest peptides, except for MifM, which stalls the ribosome at multiple consecutive codons (31). In the arrested nascent chain-ribosome complexes of these three arrest peptides, peptidyl-tRNA is located in the ribosomal P-site, suggesting that they share a basic mechanism that leads to a common mode of ribosomal stalling. The common outcome is the inhibition of further peptidyl-transfer reaction beyond the arrested P-site. Inhibition of peptidyl transfer is commonly observed among the arrest peptides in general, except for Arabidopsis CGS1, which blocks the translocation step of the elongation cycle (46).

We note that at least two different mechanisms of peptidyl-transfer inhibition are conceivable. The first possibility is that the arrest peptide somehow inhibits peptidyl-transfer from the P-site peptidyl-tRNA to the A-site amino acyl-tRNA. A structural study revealed that the ErmBL arrest peptide induces configuration changes of the PTC region of the ribosome in such a way to prevent the nucleophilic attack of the peptidyl-tRNA bond by the A-site aminoacyl-tRNA (47). The second possibility is that arrest peptide somehow interferes with the accommodation of aminoacyl-tRNA into the A-site. MifM and VemP in the stalled ribosome-nascent chain complexes were shown by cryo-EM analysis to render several ribosomal residues at the A-site to adopt an uninduced, non-productive conformation, which is incompatible with the accommodation of the aminoacyl-tRNA into the A-site (3,29–30). Molecular mechanisms of the elongation arrest by the arrest peptides identified in this study await structural analysis.

Although we did not primarily rely on the possession of a particular amino acid sequence motif in our search for ApcA, ApdA and ApdP, it turned out that they had similar amino acid sequences near their arrest sites. The R-A-P sequence is completely conserved among all the ApcA and ApdA homologs as the last three amino acid residues of the arrested peptidyl-tRNAs (Figure 1C and D). Many ApdP homologs also have R-A-P at the same position, although some ApdP homologs have glycine instead of the alanine residue (Figure 1E). The A-site codons of ApdA and ApdP are both for proline, whereas that of ApcA is for glycine, whose mutation to proline results in an enhancement of the elongation arrest. Thus, the R-A-P-P mutant form of ApcA is a better sequence for stable elongation arrest. The similarity in the arrest site-proximal sequences may either be a result of the common evolutionary origin of these arrest peptides or their convergent evolution. Their distributions in the subsets of both the gram-positive and the gram-negative species of bacteria could suggest their independent origin.

Previously, Tanner *et al.* (48) and Woolstenhulme *et al.* (49) searched into *in vitro* generated random sequence libraries for those capable of arresting translation in *E. coli* cells. They identified FxxYxIWPPP, RxPP (x can be Ser, Ala, Gly or Pro for the latter) and HGPP as elongation stallers. Although the RAPP motif in ApdA and ApdP falls into the RxPP category, ApdA does not stably arrest translation in *E. coli* (Figures 2C, 3A). Moreover, our frameshift experiments (Figure 4B, C) show that RAPP is not sufficient for the stable arrest of ApdA and ApdP in *B. subtilis* or *E. coli*. Thus, RAPP is not a sole determinant of stable elongation arrest in our cases. Woolstenhulme *et al.* (49) also showed that upstream sequences affected the arrest abilities of RxPP and HGPP. We also note that the RSPP variants of ApdA and ApdP have partially compromised the ability of elongation arrest (Figure 7B, C). Thus, the native bacterial proteins, ApdA and ApdP, must have acquired the elongation arresting properties that are tuned by the combinations of RAPP and the upstream sequences.

The essential role of the proline residues in the RAPP motif of ApdA and ApdP might be ascribed to the poor reactivity of prolyl-tRNA, either as a donor (44) or an acceptor (45), in the peptidyl transfer reaction. Recent studies highlight the translation intractability of genes encoding consecutive proline residues, typically denoted as XPPZ (X and Z are specific subsets of amino acid residues), and the overcoming function of the elongation factor-P (EF-P) (50–53). Although ApdA and ApdP have the arrest-essential di-proline, their elongation arrest takes place even in the presence of EF-P, as observed also for some other proline-rich sequences (49). Moreover, the translation arrest of ApdA and ApdP is much stronger than that mediated by some di-proline sequences. For, instance, even in the absence of EF-P, translation of YafD, which contains two di-proline (PPG) sequences, is completed within 100 s incubation *in vitro* (50). By contrast, we could hardly detect full-length species of wild type ApdA and ApdP *in vivo* even in the presence of EF-P. The EF-P-insensitive and robust elongation arrest of ApdA and ApdP suggests that the mechanism responsible for this class of new arrest peptides is distinct from the EF-P-sensitive elongation pausing at proline stretches.

Our systematic mutagenesis of the A-site residues underscored their importance in the efficient elongation arrest of the newly identified arrest peptides. In particular, proline codons are fully conserved at the A-site of ApdA and ApdP homologs (Figure 1D and E), which is required for the stable elongation arrest (Figure 8B and C). The A-site amino acid of the ApcA homologs is, in most cases, glycine and occasionally serine (Figure 1C), in accordance with the experimental result that the G104S mutation was permissive in ApcA (Figure 8A). Although we found that the G104P mutation further enhanced the elongation arrest, none of the existing ApcA homologs has proline at the A-site position. It is possible that the ApcA arrest sequence has been tuned to exhibit appropriate strength of elongation arrest (as discussed further below), as observed for the XBP1u arrest sequence (54,55). The poor reactivity of proline for peptidyl transfer (44,45,56) might generally have negative impacts on translation and, hence contribute to the programmed translation halt, including that takes place at the arrest sequences of ApdA, ApdP and the G104P mutant form of ApcA. Notably, SecM also has the essential A-site codon that specifies proline (23,24). By contrast, neither MifM nor VemP has proline at the A-site or the P-site (8,31), and VemP with the A-site proline codon has compromised ability to arrest elongation (32).

Our mutation studies show that ApcA and ApdP have a similar A-site amino acid preference. Pro is the best arrest-provoking residue for ApcA and is followed by Cys, Asn, Thr, Ser, Gly, Asp and Ala (Figure 8A). In the case of ApdP, Pro is followed by Cys, Thr, Asn, Ser, Val, Ala and Asp (Figure 8C). Of the top eight arrest-supporting A-site codons, seven are the same for ApcA and ApdP. The striking similarity in the A-site amino acid preference may suggest that ApcA and ApdP affect the PTC region of the ribosome in a similar fashion such that almost the same set of amino acids at the A-site becomes incompatible with the peptidyl transfer reaction or the aminoacyl-tRNA accommodation. By contrast, ApdA seems to have the A-site amino acid preference distinct from others (Figure 8B). The N-terminal part of the arrest sequences differs between ApcA and ApdP, suggesting that the ribosome also senses the nascent chain in the exit tunnel region in manners distinct for each arrest peptide. The A-site amino acid preference of ApcA, ApdA or ApdP seems to be distinct from that observed for the EF-P-sensitive and proline stretch-dependent stalling (49,52,57). Note that the A-site amino acid of the former corresponds to the residue after the first proline of the RAPP sequence of ApdA and ApdP, whereas that of the latter corresponds to the residue after the second proline, namely ‘Z’ of the XPPZ motif.

Previous studies of the drug-dependent ribosome stalling in the ErmAL and ErmCL regulatory peptides show that the amino acid residues at the -2 position (third from the A-site) determine the amino acid preference at the A-site for the efficient stalling induction by these peptides (58). Consistent with this observation, ApcA and ApdP share the same last three nascent chain residues, R-A-P. However, the distinct A-site amino acid preference exhibited by ApdA, which also share the R-A-P sequence, suggest that more N-terminal region of the nascent chain may also be involved in the determination of the A-site amino acid preference.

A question arises why proline is avoided at the A-site of the ApcA homologs. It is conceivable that the elongation arrest of ApcA should not be too strong for the bacterial species that possess it to survive their environments. In accordance with this assumption, the elongation arrest of ApcA seems intrinsically weaker than that of ApdA or ApdP. For instance, *in vitro* translation using *Bs* hybrid PURE system allows detection of minor full-length species of GFP-ApcA-FLAG by Western blotting (Figure 2B, lanes 1, 2, 5 and 6), while we could hardly detect the full-length products of GFP-ApdA-FLAG and GFP-ApdP-FLAG (Figure 2C and D, lanes 1, 2, 5 and 6). *In vivo*, the *B. subtilis* cells expressing *gfp-apcA-lacZ* exhibited a higher level of β-galactosidase activity than those expressing *gfp-apdA-lacZ* or *gfp-apdP-lacZ* (ex. Figure 4). We confirmed that this relatively high β-galactosidase activity was not due to an internal initiation of the *lacZ* reporter or the close proximity of GFP (a globular protein moiety) to the arrest site of ApcA (Supplementary Discussion and Figure S1).

Although the aspects of translocation-coupled arrest cancellation in ApcA, ApdA and ApdP, possible monitoring substrates, remain to be addressed experimentally, some discussion is possible by assuming that they indeed represent monitoring substrates of the respective YidC/Sec systems. Perhaps, the Sec-dependent protein translocation, a process driven by ATP, might generate a stronger pulling force than the YidC-dependent membrane insertion, which is an ATP-independent process. This difference could explain why ApcA, the putative substrate of YidC, exerts relatively weak elongation arrest as assessed by the experimental systems that are uncoupled from the membrane insertion/translocation. Our identification of the novel arrest peptides, which are conserved only in a limited range of bacterial species of vastly different phylogenetic positions, suggests that there might be far more as yet identified regulatory arrest peptides in the biological kingdoms. Different organisms may have chosen different target genes according to their specific needs determined by their inhabitation environments.

## Supplementary Material

gkab024_Supplemental_File
