# Supplementary material for: Search for translation arrest peptides encoded upstream of genes for components of protein localization pathways
Source: Nucleic Acids Res. 2021 Jan 27;49(3):1550–66. doi: 10.1093/nar/gkab024 (PMC7897499; doi:10.1093/nar/gkab024)
Supplement: gkab024_Supplemental_File [file gkab024_supplemental_file.pdf]

Supplementary Information

Supplementary Discussion

Control experiments suggesting a relatively weak elongation arrest at the ApcA arrest sequence. *B. subtilis* strain expressing *gfp-apcA-lacZ* exhibited relatively higher β-galactosidase activity than those expressing *gfp-apdA-lacZ* or *gfp-apdP-lacZ*, suggesting that the elongation arrest of ApcA is less stable than that of ApdA or ApdP. However, other explanations are possible. To address whether some internal translation initiation elevates the β-galactosidase, we mutated the initiation codon of the the *gfp* in the reporter and confirmed that the mutation reduced the β-galactosidase activity of the reporter strains to the basal levels (Supplementary Fig. S1A). Thus, it is unlikely that the higher β-galactosidase activity of the *apcA* reporter is due to an artificial internal translational initiation after the *apcA* arrest sequence. It is also conceivable that the folding of the N-terminally fused GFP might have affected the translation arrest efficiency of the arrest peptides in the context of the reporter. It was reported that folding of the Top7 protein fused N-terminally to the SecM arrest sequence leads to the arrest cancelation when the distance between Top7 and arrest site is within the range of the 31 to 38 amino acids (1). The distances between GFP and arrest sites of ApcA, ApdA, and ApdP in the reporter contexts are 43, 84, and 100 amino acids, respectively. Given that the exit tunnel of the ribosome accommodates approximately 30-40 amino acid residues, folding of GFP near the exit tunnel in the ApcA reporter could generate pulling forces that partially compromise elongation arrest of ApcA. To clarify this point, we inserted a seven-amino acids long linker (GSGGSGG) between GFP and ApcA (Supplementary Fig. 1B, left) to extend the distance between GFP and the arrest site of ApcA to 50 amino acids. β-galactosidase assay showed that the insertion of the linker only slightly lowered the levels of β-galactosidase activity of *B. subtilis* cells expressing the *gfp-apcA-lacZ* derivatives (Supplementary Fig. 1B, right). The fact that the reporter activity of the *apcA* reporter was not significantly lowered by the linker makes it unlikely that the relatively higher β-galactosidase activity of *gfp-apcA-lacZ* was due to the folding of the GFP part. Instead, it reflects an intrinsically weak translation stalling at the ApcA arrest sequence.

Reference

1. Goldman,D.H., Kaiser,C.M., Milin,A., Righini,M., Tinoco,I. and Bustamante,C. (2015) Ribosome. Mechanical force releases nascent chain-mediated ribosome arrest in vitro and in vivo. *Science*, **348**, 457–60.

Supplementary Figures

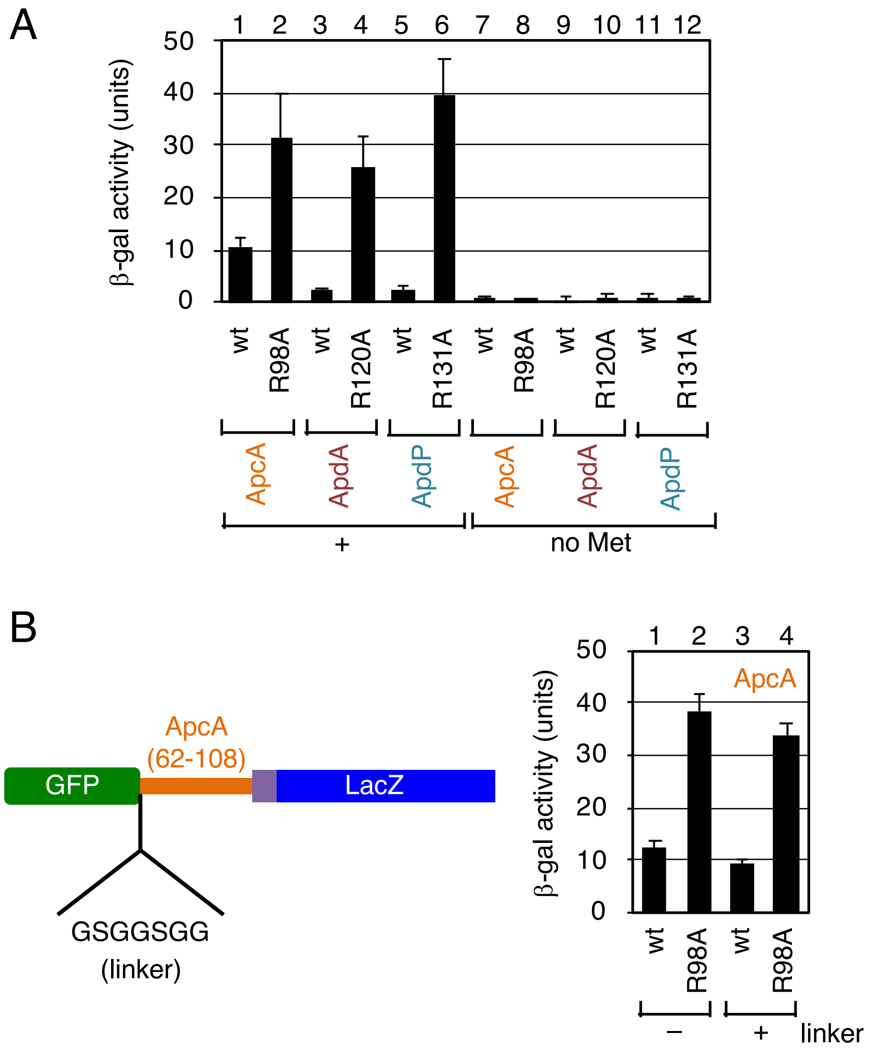

Supplementary Fig. S1. Additional controls of the *lacZ*-based translation arrest reporters. (A) Basal levels of β-galactosidase activity of the *lacZ* reporter strains used in Fig. 3C. Cell cultures of *B. subtilis* strains expressing wildtype or the arginine mutant derivatives of the *lacZ* reporters with (lanes 7-12) or without (lanes 1-6) mutation at the initiation codons for *gfp* were harvested and subjected to β-galactosidase assay (mean ± s.d., n = 4). The mutations introduced are indicated at the bottom. **(B)** Proximity of GFP to the arrest site of ApcA does not significantly impact the elongation arrest efficiency. A schematic representation of the *gfp-apcA-lacZ* reporter with a seven amino acid long linker (GSGGSGG) (left) and β-galactosidase activity of the strains expressing the *gfp-apcA-lacZ* reporter derivatives (right). Cell cultures of *B. subtilis* strains expressing wildtype or the arginine mutant derivatives of the *lacZ* reporters with (lanes 3, 4) or without (lanes 1, 2) the linker were harvested and subjected to β-galactosidase assay (mean ± s.d., n = 3).

Supplementary Table S1: List of bacterial organisms used for bioinformatics

No	taxonomy ID	Name of organisms
1	411466	Actinomyces odontolyticus ATCC 17982
2	208439	Amycolatopsis japonica
3	749927	Amycolatopsis mediterranei U32
4	1156913	Amycolatopsis orientalis HCCB10007
5	208444	Amycolatopsis vancoresmycina
6	442563	Bifidobacterium animalis subsp. lactis AD011
7	702459	Bifidobacterium bifidum PRL2010
8	326426	Bifidobacterium breve UCC2003
9	1150460	Bifidobacterium kashiwanohense JCM 15439 = DSM 21854
10	206672	Bifidobacterium longum NCC2705
11	547043	Bifidobacterium pseudocatenulatum DSM 20438 = JCM 1200 = LMG 10505
12	446465	Brachybacterium faecium DSM 4810
13	31964	Clavibacter michiganensis subsp. sepedonicus
14	525260	Corynebacterium accolens ATCC 49725
15	553204	Corynebacterium amycolatum SK46
16	257309	Corynebacterium diphtheriae NCTC 13129
17	196627	Corynebacterium glutamicum ATCC 13032
18	306537	Corynebacterium jeikeium K411
19	525264	Corynebacterium pseudogenitalium ATCC 33035
20	681645	Corynebacterium pseudotuberculosis C231
21	146827	Corynebacterium simulans
22	525268	Corynebacterium striatum ATCC 6940
23	1170318	Cutibacterium avidum 44067
24	525284	Gardnerella vaginalis ATCC 14019
25	2055	Gordonia terrae
26	1333857	Microbacterium maritypicum MF109
27	465515	Micrococcus luteus NCTC 2665
28	36809	Mycobacterium abscessus
29	262316	Mycobacterium avium subsp. paratuberculosis K-10
30	233413	Mycobacterium bovis AF2122/97
31	1774	Mycobacterium chelonae
32	1766	Mycobacterium fortuitum
33	487521	Mycobacterium intracellulare ATCC 13950
34	557599	Mycobacterium kansasii ATCC 12478
35	272631	Mycobacterium leprae TN
36	246196	Mycobacterium smegmatis str. MC2 155
37	1772278	Mycobacterium sp. GA-1331
38	83332	Mycobacterium tuberculosis H37Rv
39	1223548	Nocardia mikamii NBRC 108933
40	290340	Paenarthrobacter aurescens TC1
41	267747	Propionibacterium acnes KPA171202
42	66712	Propionibacterium freudenreichii subsp. freudenreichii
43	191292	Rhodococcus aetherivorans
44	234621	Rhodococcus erythropolis PR4
45	1051973	Rhodococcus fascians D188
46	101510	Rhodococcus jostii RHA1
47	1435356	Rhodococcus pyridinivorans SB3094
48	1278076	Rhodococcus ruber BKS 20-38
49	762948	Rothia dentocariosa ATCC 17931
50	680646	Rothia mucilaginosa DY-18
51	391037	Salinispora arenicola CNS-205
52	999542	Salinispora pacifica DSM 45543
53	369723	Salinispora tropica CNB-440
54	67257	Streptomyces albus subsp. albus
55	100226	Streptomyces coelicolor A3(2)
56	566461	Streptomyces ghanaensis ATCC 14672
57	455632	Streptomyces griseus subsp. griseus NBRC 13350
58	457428	Streptomyces lividans TK24
59	132474	Streptomyces rimosus subsp. rimosus
60	680198	Streptomyces scabiei 87.22
61	463191	Streptomyces sviceus ATCC 29083
62	953739	Streptomyces venezuelae ATCC 10712
63	1961	Streptomyces virginiae
64	203267	Tropheryma whipplei str. Twist
65	224324	Aquifex aeolicus VF5
66	309807	Salinibacter ruber DSM 13855
67	246787	Bacteroides cellulosilyticus
68	295405	Bacteroides fragilis YCH46
69	693979	Bacteroides helcogenes P 36-108
70	471870	Bacteroides intestinalis DSM 17393
71	28116	Bacteroides ovatus
72	226186	Bacteroides thetaiotaomicron VPI-5482
73	997889	Bacteroides uniformis CL03T00C23

74	435590	Bacteroides vulgatus ATCC 8482
75	435591	Parabacteroides distasonis ATCC 8503
76	431947	Porphyromonas gingivalis ATCC 33277
77	553174	Prevotella melaninogenica ATCC 25845
78	521097	Capnocytophaga ochracea DSM 7271
79	525257	Chryseobacterium gleum ATCC 35910
80	253	Chryseobacterium indologenes
81	1338011	Elizabethkingia anophelis NUHP1
82	362413	Flavobacterium aquidurense
83	402612	Flavobacterium psychrophilum JIP02/86
84	362418	Flavobacterium reichenbachii
85	76832	Myroides odoratimimus
86	693978	Riemerella anatipestifer ATCC 11845 = DSM 15868
87	1454007	Pedobacter borealis DSM 19626
88	243161	Chlamydia muridarum str. Nigg
89	331636	Chlamydia psittaci 6BC
90	471472	Chlamydia trachomatis 434/Bu
91	331635	Chlamydophila pecorum E58
92	115713	Chlamydophila pneumoniae CWL029
93	194439	Chlorobium tepidum TLS
94	324602	Chloroflexus aurantiacus J-10-fl
95	243164	Dehalococcoides mccartyi 195
96	93060	Prochlorococcus marinus str. MIT 9215
97	197221	Thermosynechococcus elongatus BP-1
98	251221	Gloeobacter violaceus PCC 7421
99	449447	Microcystis aeruginosa NIES-843
100	243230	Deinococcus radiodurans R1
101	300852	Thermus thermophilus HB8
102	515635	Dictyoglomus turgidum DSM 6724
103	491915	Anoxybacillus flavithermus WK1
104	198467	Anoxybacillus gonensis
105	692420	Bacillus amyloliquefaciens DSM 7
106	198094	Bacillus anthracis str. Ames
107	1529886	Bacillus atrophaeus subsp. globigii
108	226900	Bacillus cereus ATCC 14579
109	345219	Bacillus coagulans 36D1
110	279010	Bacillus licheniformis DSM 13 = ATCC 14580
111	592022	Bacillus megaterium DSM 319
112	526998	Bacillus mycoides Rock1-4
113	1408	Bacillus pumilus
114	1478	Bacillus simplex
115	224308	Bacillus subtilis subsp. subtilis str. 168
116	529122	Bacillus thuringiensis YBT-1518
117	358681	Brevibacillus brevis NBRC 100599
118	1121126	Brochothrix thermosphacta DSM 20171 = FSL F6-1036
119	565655	Enterococcus casseliflavus EC20
120	1121864	Enterococcus cecorum DSM 20682 = ATCC 43198
121	226185	Enterococcus faecalis V583
122	1158608	Enterococcus haemoperoxidus ATCC BAA-382
123	768486	Enterococcus hirae ATCC 9790
124	1397697	Exiguobacterium acetylicum DSM 20416
125	235909	Geobacillus kaustophilus HTA426
126	272621	Lactobacillus acidophilus NCFM
127	1423821	Lactobacillus aviarius subsp. aviarius DSM 20655
128	387344	Lactobacillus brevis ATCC 367
129	748671	Lactobacillus crispatus ST1
130	390333	Lactobacillus delbrueckii subsp. bulgaricus ATCC 11842 = JCM 1002
131	334390	Lactobacillus fermentum IFO 3956
132	324831	Lactobacillus gasseri ATCC 33323 = JCM 1131
133	326425	Lactobacillus helveticus CNRZ32
134	525328	Lactobacillus iners DSM 13335
135	525329	Lactobacillus jensenii JV-V16
136	257314	Lactobacillus johnsonii NCC 533
137	321967	Lactobacillus paracasei ATCC 334
138	220668	Lactobacillus plantarum WCFS1
139	557436	Lactobacillus reuteri DSM 20016
140	568703	Lactobacillus rhamnosus GG
141	1069534	Lactobacillus ruminis ATCC 27782
142	314315	Lactobacillus sakei subsp. sakei 23K
143	362948	Lactobacillus salivarius UCC118
144	420890	Lactococcus garvieae Lg2
145	272623	Lactococcus lactis subsp. lactis Il1403
146	203120	Leuconostoc mesenteroides subsp. mesenteroides ATCC 8293
147	169963	Listeria monocytogenes EGD-e
148	28031	Lysinibacillus fusiformis
149	940190	Melissococcus plutonius ATCC 35311

150	203123	Oenococcus oeni PSU-1
151	160799	Paenibacillus borealis
152	886882	Paenibacillus polymyxa SC2
153	1426	Parageobacillus thermoglucosidans
154	862514	Pediococcus acidilactici DSM 20284
155	93061	Staphylococcus aureus subsp. aureus NCTC 8325
156	1296619	Staphylococcus capitis CR01
157	176280	Staphylococcus epidermidis ATCC 12228
158	246432	Staphylococcus equorum
159	279808	Staphylococcus haemolyticus JCSC1435
160	435837	Staphylococcus hominis subsp. hominis C80
161	698737	Staphylococcus lugdunensis HKU09-01
162	937773	Staphylococcus pseudintermedius HKU10-03
163	342451	Staphylococcus saprophyticus subsp. saprophyticus ATCC 15305
164	147467	Staphylococcus sciuri subsp. sciuri
165	1194526	Staphylococcus warneri SG1
166	1288	Staphylococcus xylosus
167	208435	Streptococcus agalactiae 2603V/R
168	862971	Streptococcus anginosus C238
169	1302863	Streptococcus cristatus AS 1.3089
170	759913	Streptococcus dysgalactiae subsp. equisimilis AC-2713
171	40041	Streptococcus equi subsp. zooepidemicus
172	981539	Streptococcus gallolyticus subsp. gallolyticus ATCC 43143
173	467705	Streptococcus gordonii str. Challis substr. CH1
174	889204	Streptococcus infantis ATCC 700779
175	862967	Streptococcus intermedius B196
176	365659	Streptococcus mitis B6
177	210007	Streptococcus mutans UA159
178	760570	Streptococcus parasanguinis ATCC 15912
179	936154	Streptococcus parauberis KCTC 11537
180	888746	Streptococcus peroris ATCC 700780
181	171101	Streptococcus pneumoniae R6
182	160490	Streptococcus pyogenes M1 GAS
183	1048332	Streptococcus salivarius CCHSS3
184	388919	Streptococcus sanguinis SK36
185	1123317	Streptococcus sobrinus DSM 20742 = ATCC 33478
186	568814	Streptococcus suis BM407
187	264199	Streptococcus thermophilus LMG 18311
188	218495	Streptococcus uberis 0140J
189	1121115	Blautia wexlerae DSM 19850
190	272563	Clostridioides difficile 630
191	272562	Clostridium acetobutylicum ATCC 824
192	997894	Clostridium bolteae 90A9
193	413999	[Clostridium] botulinum A str. ATCC 3502
194	997898	Clostridium butyricum 60E.3
195	386415	Clostridium novyi NT
196	195103	Clostridium perfringens ATCC 13124
197	212717	Clostridium tetani E88
198	1519	Clostridium tyrobutyricum
199	658086	Lachnospiraceae bacterium 3_1_57FAA_CT1
200	264732	Moorella thermoacetica ATCC 39073
201	1292035	Paeniclostridium sordellii VPI 9048
202	697303	Thermoanaerobacter wiegelii Rt8.B1
203	999413	[Clostridium] innocuum 2959
204	479436	Veillonella parvula DSM 2008
205	1410656	Fusobacterium necrophorum HUN048
206	190304	Fusobacterium nucleatum subsp. nucleatum ATCC 25586
207	519441	Streptobacillus moniliformis DSM 12112
208	289376	Thermodesulfovibrio yellowstonii DSM 11347
209	243090	Rhodopirellula baltica SH 1
210	637390	Acidithiobacillus thiooxidans ATCC 19377
211	178901	Acetobacter malorum
212	634452	Acetobacter pasteurianus IFO 3283-01
213	883078	Afipia broomeae ATCC 49717
214	176299	Agrobacterium fabrum str. C58
215	311403	Agrobacterium radiobacter K84
216	358	Agrobacterium tumefaciens
217	320483	Anaplasma marginale str. Florida
218	212042	Anaplasma phagocytophilum str. HZ
219	360095	Bartonella bacilliformis KC583
220	283166	Bartonella henselae str. Houston-1
221	1225179	Bartonella quintana RM-11
222	224911	Bradyrhizobium diazoefficiens USDA 110
223	398525	Bradyrhizobium elkanii USDA 76
224	936455	Bradyrhizobium genosp. SA-4 str. CB756
225	1037409	Bradyrhizobium japonicum USDA 6

226	359391	Brucella abortus 2308
227	224914	Brucella melitensis bv. 1 str. 16M
228	190650	Caulobacter crescentus CB15
229	1416753	Ensifer adhaerens OV14
230	290633	Gluconobacter oxydans 621H
231	759362	Ketogulonicigenium vulgare WSH-001
232	1296990	Komagataeibacter xylinus E25
233	1082933	Mesorhizobium amorphae CCNWGS0123
234	765698	Mesorhizobium ciceri biovar biserrulae WSM1271
235	266835	Mesorhizobium loti MAFF303099
236	272630	Methylobacterium extorquens AM1
237	426355	Methylobacterium radiotolerans JCM 2831
238	1028800	Neorhizobium galegae bv. orientalis str. HAMBI 540
239	439375	Ochrobactrum anthropi ATCC 49188
240	357244	Orientia tsutsugamushi str. Boryong
241	989396	Pseudovibrio sp. Ad13
242	347834	Rhizobium etli CFN 42
243	1033991	Rhizobium leguminosarum bv. trifolii CB782
244	272943	Rhodobacter sphaeroides 2.4.1
245	269796	Rhodospirillum rubrum ATCC 11170
246	272944	Rickettsia conorii str. Malish 7
247	272947	Rickettsia prowazekii str. Madrid E
248	1265309	Ruegeria mobilis F1926
249	394	Sinorhizobium fredii NGR234
250	366394	Sinorhizobium medicae WSM419
251	266834	Sinorhizobium meliloti 1021
252	452662	Sphingobium japonicum UT26S
253	1219050	Sphingomonas paucimobilis NBRC 13935
254	1736216	Sphingomonas sp. Leaf34
255	1515612	Sphingopyxis fribergensis
256	163164	Wolbachia endosymbiont of Drosophila melanogaster
257	762376	Achromobacter xylosoxidans A8
258	511	Alcaligenes faecalis
259	568707	Bordetella bronchiseptica 253
260	1331262	Bordetella hinzii OH87 BAL007II
261	1247649	Bordetella holmesii ATCC 51541
262	1208660	Bordetella parapertussis Bpp5
263	257313	Bordetella pertussis Tohama I
264	983594	Burkholderia cepacia ATCC 25416
265	28095	Burkholderia gladioli
266	626418	Burkholderia glumae BGR1
267	243160	Burkholderia mallei ATCC 23344
268	87883	Burkholderia multivorans
269	272560	Burkholderia pseudomallei K96243
270	1249668	Burkholderia ubonensis MSMB22
271	871203	Caballeronia zhejiangensis
272	243365	Chromobacterium violaceum ATCC 12472
273	688245	Comamonas testosteroni CNB-2
274	266264	Cupriavidus metallidurans CH34
275	977880	Cupriavidus taiwanensis LMG 19424
276	398578	Delftia acidovorans SPH-1
277	546274	Eikenella corrodens ATCC 23834
278	757424	Herbaspirillum seropedicae SmR1
279	1349767	Janthinobacterium agaricidamnosum NBRC 102515 = DSM 9628
280	887327	Kingella kingae ATCC 23330
281	242231	Neisseria gonorrhoeae FA 1090
282	489653	Neisseria lactamica 020-06
283	122586	Neisseria meningitidis MC58
284	435832	Neisseria mucosa C102
285	547045	Neisseria sicca ATCC 29256
286	93220	Pandoraea pnomenusa
287	1229205	Paraburkholderia phenoliruptrix BR3459a
288	391038	Paraburkholderia phymatum STM815
289	266265	Paraburkholderia xenovorans LB400
290	381666	Ralstonia eutropha H16
291	402626	Ralstonia pickettii 12J
292	267608	Ralstonia solanacearum GMI1000
293	595537	Variovorax paradoxus EPS
294	882	Desulfovibrio vulgaris str. Hildenborough
295	243231	Geobacter sulfurreducens PCA
296	367737	Arcobacter butzleri RM4018
297	1183379	Campylobacter coli RM4661
298	199	Campylobacter concisus
299	360106	Campylobacter fetus subsp. fetus 82-40
300	192222	Campylobacter jejuni subsp. jejuni NCTC 11168 = ATCC 700819
301	306263	Campylobacter lari RM2100

302	1388752	Campylobacter subantarcticus LMG 24377
303	537972	Helicobacter pullorum MIT 98-5489
304	85962	Helicobacter pylori 26695
305	1120924	Acinetobacter baylyi DSM 14961 = CIP 107474
306	1217648	Acinetobacter beijerinckii CIP 110307
307	981324	Acinetobacter bereziniae LMG 1003 = CIP 70.12
308	1217658	Acinetobacter gyllenbergii NIPH 230
309	1217659	Acinetobacter haemolyticus CIP 64.3
310	1242245	Acinetobacter johnsonii XBB1
311	1217664	Acinetobacter junii CIP 64.5
312	981327	Acinetobacter lwoffii NCTC 5866 = CIP 64.10
313	436717	Acinetobacter oleivorans DR1
314	981333	Acinetobacter parvus DSM 16617 = CIP 108168
315	871585	Acinetobacter pittii PHEA-2
316	981334	Acinetobacter radioresistens DSM 6976 = NBRC 102413 = CIP 103788
317	1217988	Acinetobacter schindleri CIP 107287
318	1217677	Acinetobacter soli NIPH 2899
319	981336	Acinetobacter ursingii DSM 16037 = CIP 107286
320	1197884	Acinetobacter venetianus VE-C3
321	416269	Actinobacillus pleuropneumoniae serovar 5b str. L20
322	380703	Aeromonas hydrophila subsp. hydrophila ATCC 7966
323	382245	Aeromonas salmonicida subsp. salmonicida A449
324	654	Aeromonas veronii
325	1407647	Aggregatibacter actinomycetemcomitans NUM4039
326	80852	Aliivibrio wodanis
327	529120	Alteromonas macleodii ATCC 27126
328	1774373	Alteromonas mediterranea DE
329	107806	Buchnera aphidicola str. APS (Acyrthosiphon pisum)
330	1333848	Citrobacter freundii CFNIH1
331	227377	Coxiella burnetii RSA 493
332	28141	Cronobacter sakazakii
333	198628	Dickeya dadantii 3937
334	1225786	Dickeya solani IPO 2222
335	634503	Edwardsiella ictaluri 93-146
336	1028307	[Enterobacter] aerogenes KCTC 2190
337	716541	Enterobacter cloacae subsp. cloacae ATCC 13047
338	665029	Erwinia amylovora CFBP1430
339	511145	Escherichia coli str. K-12 substr. MG1655
340	484022	Francisella philomiragia subsp. philomiragia ATCC 25017
341	177416	Francisella tularensis subsp. tularensis SCHU S4
342	1005058	Gallibacterium anatis UMN179
343	1196095	Gilliamella apicola
344	233412	Haemophilus ducreyi 35000HP
345	71421	Haemophilus influenzae Rd KW20
346	862965	Haemophilus parainfluenzae T3T1
347	557723	Haemophilus parasuis SH0165
348	1072583	Halomonas boliviensis LC1
349	571	Klebsiella oxytoca
350	1125630	Klebsiella pneumoniae subsp. pneumoniae HS11286
351	1235834	Kosakonia sacchari SP1
352	83655	Leclercia adecarboxylata
353	272624	Legionella pneumophila subsp. pneumophila str. Philadelphia 1
354	1316932	Mannheimia haemolytica M42548
355	1236608	Moraxella catarrhalis BBH18
356	1124991	Morganella morganii subsp. morganii KT
357	82983	Obesumbacterium proteus
358	549	Pantoea agglomerans
359	1123863	Pantoea ananatis LMG 5342
360	1358411	Pantoea dispersa EGD-AAK13
361	1076550	Pantoea rwandensis
362	272843	Pasteurella multocida subsp. multocida str. Pm70
363	218491	Pectobacterium atrosepticum SCRI1043
364	561230	Pectobacterium carotovorum subsp. carotovorum PC1
365	1001530	Photobacterium leiognathi subsp. mandapamensis svers.1.1.
366	1454202	Photobacterium phosphoreum ANT-2200
367	230089	Photorhabdus temperata subsp. thracensis
368	1227812	Piscirickettsia salmonis LF-89 = ATCC VR-1361
369	61647	Pluralibacter gergoviae
370	529507	Proteus mirabilis HI4320
371	1141663	Providencia rettgeri Dmel1
372	326442	Pseudoalteromonas haloplanktis TAC125
373	152297	Pseudoalteromonas issachenkonii
374	1353533	Pseudoalteromonas luteoviolacea 2ta16
375	1190813	Pseudoalteromonas shioyasakiensis
376	162160	Pseudoalteromonas telluritireducens
377	1117320	Pseudoalteromonas undina DSM 6065

378	208964	Pseudomonas aeruginosa PAO1
379	237609	Pseudomonas alkylphenolica
380	1114970	Pseudomonas fluorescens F113
381	1136138	Pseudomonas fragi B25
382	1301098	Pseudomonas knackmussii B13
383	399739	Pseudomonas mendocina ymp
384	47885	Pseudomonas oryzihabitans
385	1124983	Pseudomonas protegens CHA0
386	160488	Pseudomonas putida KT2440
387	379731	Pseudomonas stutzeri A1501
388	205918	Pseudomonas syringae pv. syringae B728a
389	1122936	Rodentibacter pneumotropicus DSM 21403
390	220341	Salmonella enterica subsp. enterica serovar Typhi str. CT18
391	47917	Serratia fonticola
392	1346614	Serratia liquefaciens ATCC 27592
393	273526	Serratia marcescens subsp. marcescens Db11
394	1154756	Serratia plymuthica PRI-2C
395	693973	Shewanella baltica OS678
396	211586	Shewanella oneidensis MR-1
397	300267	Shigella dysenteriae Sd197
398	198214	Shigella flexneri 2a str. 301
399	522373	Stenotrophomonas maltophilia K279a
400	377629	Teredinibacter turnerae T7901
401	396595	Thioalkalivibrio sp. K90mix
402	106634	Thioalkalivibrio versutus
403	1219076	Vibrio alginolyticus NBRC 15630 = ATCC 17749
404	882102	Vibrio anguillarum 775
405	945543	Vibrio brasiliensis LMG 20546
406	338187	Vibrio campbellii ATCC BAA-1116
407	243277	Vibrio cholerae O1 biovar El Tor str. N16961
408	190893	Vibrio coralliilyticus
409	1136163	Vibrio cyclitrophicus FF75
410	312309	Vibrio fischeri ES114
411	676	Vibrio fluvialis
412	28173	Vibrio nigripulchritudo
413	223926	Vibrio parahaemolyticus RIMD 2210633
414	575788	Vibrio tasmaniensis LGP32
415	1051646	Vibrio tubiashii ATCC 19109
416	196600	Vibrio vulnificus YJ016
417	380358	Xanthomonas albilineans GPE PC73
418	56448	Xanthomonas arboricola
419	1304892	Xanthomonas axonopodis Xac29-1
420	190485	Xanthomonas campestris pv. campestris str. ATCC 33913
421	291331	Xanthomonas oryzae pv. oryzae KACC 10331
422	487909	Xanthomonas translucens pv. undulosa
423	406818	Xenorhabdus bovienii SS-2004
424	160492	Xylella fastidiosa 9a5c
425	393305	Yersinia enterocolitica subsp. enterocolitica 8081
426	214092	Yersinia pestis CO92
427	29486	Yersinia ruckeri
428	224326	Borrelia burgdorferi B31
429	1234596	Borrelia garinii NMJW1
430	565034	Brachyspira hyodysenteriae WA1
431	355276	Leptospira borgpetersenii serovar Hardjo-bovis str. L550
432	189518	Leptospira interrogans serovar Lai str. 56601
433	1049968	Leptospira kirschneri serovar Valbuzzi str. 200702274
434	1001595	Leptospira noguchii serovar Panama str. CZ214
435	758847	Leptospira santarosai serovar Shermani str. LT 821
436	1088540	Leptospira weilii serovar Topaz str. LT2116
437	243275	Treponema denticola ATCC 35405
438	243276	Treponema pallidum subsp. pallidum str. Nichols
439	525903	Thermanaerovibrio acidaminovorans DSM 6589
440	265311	Mesoplasma florum L1
441	340047	Mycoplasma capricolum subsp. capricolum ATCC 27343
442	710127	Mycoplasma gallisepticum str. R(low)
443	347256	Mycoplasma hominis ATCC 23114
444	272632	Mycoplasma mycoides subsp. mycoides SC str. PG1
445	1446495	Mycoplasma ovipneumoniae NM2010
446	272634	Mycoplasma pneumoniae M129
447	505682	Ureaplasma parvum serovar 3 str. ATCC 27815
448	243274	Thermotoga maritima MSB8
449	390874	Thermotoga petrophila RKU-1

Supplementary Table S2: *B. subtilis* strains used in this study

Strains	Genotype	Plasmid	Parent	ref
PY79	wild type			1
SCB610	*ybaC Ω kan*			2
SCB3958	*amyE::PmifM gfp-apcA-flag-lacZ Ω cat*	pCH2124	PY79	This study
SCB3959	*amyE::PmifM gfp-apdA-flag-lacZ Ω cat*	pCH2125	PY79	This study
SCB3960	*amyE::PmifM gfp-apdP-flag-lacZ Ω cat*	pCH2126	PY79	This study
SCB3961	*amyE::PmifM gfp-apcA(R98A)-flag-lacZ Ω cat*	pCH2127	PY79	This study
SCB3962	*amyE::PmifM gfp-apdP(R131A)-flag-lacZ Ω cat*	pCH2128	PY79	This study
SCB3967	*amyE::PmifM gfp-apcA62-flag Ω cat, ybaC Ω kan*	pCH2105	SCB610	This study
SCB3968	*amyE::PmifM gfp-apdA39-flag Ω cat, ybaC Ω kan*	pCH2107	SCB610	This study
SCB3969	*amyE::PmifM gfp-apdP34-flag Ω cat, ybaC Ω kan*	pCH2108	SCB610	This study
SCB3970	*amyE::PmifM gfp-apcA62(R98A)-flag Ω cat, ybaC Ω kan*	pCH2118	SCB610	This study
SCB3971	*amyE::PmifM gfp-apdA39(R120A)-flag Ω cat, ybaC Ω kan*	pCH2120	SCB610	This study
SCB3972	*amyE::PmifM gfp-apdP34(R131A)-flag Ω cat, ybaC Ω kan*	pCH2122	SCB610	This study
SCB3999	*amyE::PmifM gfp-apdA(R120A)-flag-lacZ Ω cat*	pCH2134	PY79	This study
SCB4032	*amyE::PmifM gfp-apcA(G105A)-flag-lacZ Ω cat*	pCH2162	PY79	This study
SCB4033	*amyE::PmifM gfp-apcA(G105L)-flag-lacZ Ω cat*	pCH2163	PY79	This study
SCB4034	*amyE::PmifM gfp-apcA(G105R)-flag-lacZ Ω cat*	pCH2164	PY79	This study
SCB4036	*amyE::PmifM gfp-apcA(G105E)-flag-lacZ Ω cat*	pCH2166	PY79	This study
SCB4037	*amyE::PmifM gfp-apcA(G105W)-flag-lacZ Ω cat*	pCH2167	PY79	This study
SCB4038	*amyE::PmifM gfp-apcA(G105S)-flag-lacZ Ω cat*	pCH2168	PY79	This study
SCB4039	*amyE::PmifM gfp-apcA(G105Y)-flag-lacZ Ω cat*	pCH2169	PY79	This study
SCB4040	*amyE::PmifM gfp-apcA(G105P)-flag-lacZ Ω cat*	pCH2170	PY79	This study
SCB4041	*amyE::PmifM gfp-apcA(G105M)-flag-lacZ Ω cat*	pCH2171	PY79	This study
SCB4042	*amyE::PmifM gfp-apcA(G105V)-flag-lacZ Ω cat*	pCH2172	PY79	This study
SCB4043	*amyE::PmifM gfp-apcA(G105C)-flag-lacZ Ω cat*	pCH2173	PY79	This study
SCB4044	*amyE::PmifM gfp-apcA(G105D)-flag-lacZ Ω cat*	pCH2174	PY79	This study
SCB4045	*amyE::PmifM gfp-apdA(P123V)-flag-lacZ Ω cat*	pCH2175	PY79	This study
SCB4046	*amyE::PmifM gfp-apdA(P123A)-flag-lacZ Ω cat*	pCH2176	PY79	This study
SCB4047	*amyE::PmifM gfp-apdA(P123G)-flag-lacZ Ω cat*	pCH2177	PY79	This study
SCB4048	*amyE::PmifM gfp-apdA(P123N)-flag-lacZ Ω cat*	pCH2178	PY79	This study
SCB4049	*amyE::PmifM gfp-apdA(P123I)-flag-lacZ Ω cat*	pCH2179	PY79	This study
SCB4050	*amyE::PmifM gfp-apdA(P123S)-flag-lacZ Ω cat*	pCH2181	PY79	This study
SCB4052	*amyE::PmifM gfp-apdA(P123R)-flag-lacZ Ω cat*	pCH2183	PY79	This study
SCB4053	*amyE::PmifM gfp-apdA(P123F)-flag-lacZ Ω cat*	pCH2184	PY79	This study
SCB4054	*amyE::PmifM gfp-apdA(P123W)-flag-lacZ Ω cat*	pCH2185	PY79	This study
SCB4055	*amyE::PmifM gfp-apdA(P123K)-flag-lacZ Ω cat*	pCH2186	PY79	This study
SCB4056	*amyE::PmifM gfp-apdA(P123H)-flag-lacZ Ω cat*	pCH2187	PY79	This study
SCB4057	*amyE::PmifM gfp-apdA(P123C)-flag-lacZ Ω cat*	pCH2188	PY79	This study
SCB4058	*amyE::PmifM gfp-apdA(P123D)-flag-lacZ Ω cat*	pCH2189	PY79	This study
SCB4059	*amyE::PmifM gfp-apdA(P123L)-flag-lacZ Ω cat*	pCH2190	PY79	This study
SCB4060	*amyE::PmifM gfp-apdA(P123T)-flag-lacZ Ω cat*	pCH2191	PY79	This study
SCB4061	*amyE::PmifM gfp-apdA(P123M)-flag-lacZ Ω cat*	pCH2192	PY79	This study
SCB4063	*amyE::PmifM gfp-apdP(P134A)-flag-lacZ Ω cat*	pCH2194	PY79	This study
SCB4064	*amyE::PmifM gfp-apdP(P134Q)-flag-lacZ Ω cat*	pCH2195	PY79	This study
SCB4065	*amyE::PmifM gfp-apdP(P134T)-flag-lacZ Ω cat*	pCH2196	PY79	This study
SCB4066	*amyE::PmifM gfp-apdP(P134D)-flag-lacZ Ω cat*	pCH2197	PY79	This study
SCB4067	*amyE::PmifM gfp-apdP(P134L)-flag-lacZ Ω cat*	pCH2198	PY79	This study
SCB4068	*amyE::PmifM gfp-apdP(P134V)-flag-lacZ Ω cat*	pCH2199	PY79	This study
SCB4069	*amyE::PmifM gfp-apdP(P134F)-flag-lacZ Ω cat*	pCH2200	PY79	This study
SCB4070	*amyE::PmifM gfp-apdP(P134S)-flag-lacZ Ω cat*	pCH2201	PY79	This study
SCB4071	*amyE::PmifM gfp-apdP(P134G)-flag-lacZ Ω cat*	pCH2202	PY79	This study
SCB4072	*amyE::PmifM gfp-apdP(P134R)-flag-lacZ Ω cat*	pCH2203	PY79	This study

SCB4073	amyE::PmifM gfp-apdP(P134W)-flag-lacZ Ω cat	pCH2204	PY79	This study
SCB4074	amyE::PmifM gfp-apdP(P134Y)-flag-lacZ Ω cat	pCH2205	PY79	This study
SCB4075	amyE::PmifM gfp-apcA(G105F)-flag-lacZ Ω cat	pCH2206	PY79	This study
SCB4076	amyE::PmifM gfp-apcA(G105T)-flag-lacZ Ω cat	pCH2207	PY79	This study
SCB4077	amyE::PmifM gfp-apcA(G105N)-flag-lacZ Ω cat	pCH2208	PY79	This study
SCB4078	amyE::PmifM gfp-apcA(G105K)-flag-lacZ Ω cat	pCH2209	PY79	This study
SCB4079	amyE::PmifM gfp-apcA(G105H)-flag-lacZ Ω cat	pCH2210	PY79	This study
SCB4080	amyE::PmifM gfp-apcA(G105I)-flag-lacZ Ω cat	pCH2211	PY79	This study
SCB4081	amyE::PmifM gfp-apcA(G105Q)-flag-lacZ Ω cat	pCH2212	PY79	This study
SCB4082	amyE::PmifM gfp-apdA(P123Y)-flag-lacZ Ω cat	pCH2213	PY79	This study
SCB4083	amyE::PmifM gfp-apdA(P123Q)-flag-lacZ Ω cat	pCH2214	PY79	This study
SCB4084	amyE::PmifM gfp-apdA(P123E)-flag-lacZ Ω cat	pCH2215	PY79	This study
SCB4085	amyE::PmifM gfp-apdP(P134I)-flag-lacZ Ω cat	pCH2216	PY79	This study
SCB4086	amyE::PmifM gfp-apdP(P134K)-flag-lacZ Ω cat	pCH2217	PY79	This study
SCB4087	amyE::PmifM gfp-apdP(P134N)-flag-lacZ Ω cat	pCH2218	PY79	This study
SCB4088	amyE::PmifM gfp-apdP(P134C)-flag-lacZ Ω cat	pCH2219	PY79	This study
SCB4089	amyE::PmifM gfp-apdP(P134M)-flag-lacZ Ω cat	pCH2220	PY79	This study
SCB4090	amyE::PmifM gfp-apdP(P134H)-flag-lacZ Ω cat	pCH2221	PY79	This study
SCB4091	amyE::PmifM gfp-apdP(P134E)-flag-lacZ Ω cat	pCH2222	PY79	This study
SKB5	amyE::PmifM gfp-apcA_FS62-108-flag-lacZ Ω cat	pSK6	PY79	This study
SKB6	amyE::PmifM gfp-apcA_FS62-98-flag-lacZ Ω cat	pSK8	PY79	This study
SKB7	amyE::PmifM gfp-apcA_FS62-88-flag-lacZ Ω cat	pSK9	PY79	This study
SKB8	amyE::PmifM gfp-apcA_FS62-78-flag-lacZ Ω cat	pSK13	PY79	This study
SKB9	amyE::PmifM gfp-apdP_FS34-140-flag-lacZ Ω cat	pSK14	PY79	This study
SKB11	amyE::PmifM gfp-apdP_FS34-130-flag-lacZ Ω cat	pSK16	PY79	This study
SKB13	amyE::PmifM gfp-apdP_FS34-120-flag-lacZ Ω cat	pSK18	PY79	This study
SKB15	amyE::PmifM gfp-apdP_FS34-110-flag-lacZ Ω cat	pSK20	PY79	This study
SKB17	amyE::PmifM gfp-apcA_FS62-73-flag-lacZ Ω cat	pSK30	PY79	This study
SKB19	amyE::PmifM gfp-apdP_FS34-125-flag-lacZ Ω cat	pSK24	PY79	This study
SKB25	amyE::PmifM gfp-apcA(T74A)-flag-lacZ Ω cat	pSK38	PY79	This study
SKB26	amyE::PmifM gfp-apcA(P76A)-flag-lacZ Ω cat	pSK40	PY79	This study
SKB27	amyE::PmifM gfp-apcA(S77A)-flag-lacZ Ω cat	pSK41	PY79	This study
SKB28	amyE::PmifM gfp-apcA(D78A)-flag-lacZ Ω cat	pSK42	PY79	This study
SKB29	amyE::PmifM gfp-apcA(D79A)-flag-lacZ Ω cat	pSK43	PY79	This study
SKB30	amyE::PmifM gfp-apcA(G75A)-flag-lacZ Ω cat	pSK39	PY79	This study
SKB31	amyE::PmifM gfp-apcA(C82A)-flag-lacZ Ω cat	pSK46	PY79	This study
SKB32	amyE::PmifM gfp-apcA(L83A)-flag-lacZ Ω cat	pSK47	PY79	This study
SKB33	amyE::PmifM gfp-apcA(R84A)-flag-lacZ Ω cat	pSK48	PY79	This study
SKB34	amyE::PmifM gfp-apcA(G85A)-flag-lacZ Ω cat	pSK49	PY79	This study
SKB35	amyE::PmifM gfp-apcA(A86S)-flag-lacZ Ω cat	pSK50	PY79	This study
SKB36	amyE::PmifM gfp-apcA(F87A)-flag-lacZ Ω cat	pSK51	PY79	This study
SKB37	amyE::PmifM gfp-apcA(R88A)-flag-lacZ Ω cat	pSK52	PY79	This study
SKB38	amyE::PmifM gfp-apcA(R89A)-flag-lacZ Ω cat	pSK53	PY79	This study
SKB39	amyE::PmifM gfp-apcA(R81A)-flag-lacZ Ω cat	pSK45	PY79	This study
SKB40	amyE::PmifM gfp-apcA(S91A)-flag-lacZ Ω cat	pSK55	PY79	This study
SKB41	amyE::PmifM gfp-apcA(P93A)-flag-lacZ Ω cat	pSK58	PY79	This study
SKB42	amyE::PmifM gfp-apcA(D94A)-flag-lacZ Ω cat	pSK59	PY79	This study
SKB43	amyE::PmifM gfp-apcA(G97A)-flag-lacZ Ω cat	pSK60	PY79	This study
SKB44	amyE::PmifM gfp-apcA(E80A)-flag-lacZ Ω cat	pSK44	PY79	This study
SKB45	amyE::PmifM gfp-apcA(Q90A)-flag-lacZ Ω cat	pSK54	PY79	This study
SKB46	amyE::PmifM gfp-apcA(S92A)-flag-lacZ Ω cat	pSK56	PY79	This study
SKB47	amyE::PmifM gfp-apcA(A95S)-flag-lacZ Ω cat	pSK57	PY79	This study
SKB48	amyE::PmifM gfp-apcA(P96A)-flag-lacZ Ω cat	pSK67	PY79	This study
SKB49	amyE::PmifM gfp-apcA(P99A)-flag-lacZ Ω cat	pSK61	PY79	This study

SKB50	*amyE::PmifM gfp-apcA(Q100A)-flag-lacZ Ω cat*	pSK65	PY79	This study
SKB51	*amyE::PmifM gfp-apcA(P101A)-flag-lacZ Ω cat*	pSK62	PY79	This study
SKB52	*amyE::PmifM gfp-apcA(R102A)-flag-lacZ Ω cat*	pSK63	PY79	This study
SKB53	*amyE::PmifM gfp-apcA(A103S)-flag-lacZ Ω cat*	pSK64	PY79	This study
SKB54	*amyE::PmifM gfp-apcA(P104A)-flag-lacZ Ω cat*	pSK66	PY79	This study
SKB62	*amyE::PmifM gfp-apdA(S109A)-flag-lacZ Ω cat*	pSK76	PY79	This study
SKB63	*amyE::PmifM gfp-apdA(R110A)-flag-lacZ Ω cat*	pSK77	PY79	This study
SKB64	*amyE::PmifM gfp-apdA(A112S)-flag-lacZ Ω cat*	pSK79	PY79	This study
SKB65	*amyE::PmifM gfp-apdA(N113A)-flag-lacZ Ω cat*	pSK80	PY79	This study
SKB66	*amyE::PmifM gfp-apdA(R114A)-flag-lacZ Ω cat*	pSK81	PY79	This study
SKB67	*amyE::PmifM gfp-apdA(L115A)-flag-lacZ Ω cat*	pSK82	PY79	This study
SKB68	*amyE::PmifM gfp-apdA(A116S)-flag-lacZ Ω cat*	pSK83	PY79	This study
SKB69	*amyE::PmifM gfp-apdA(L117A)-flag-lacZ Ω cat*	pSK84	PY79	This study
SKB70	*amyE::PmifM gfp-apdA(G118A)-flag-lacZ Ω cat*	pSK85	PY79	This study
SKB71	*amyE::PmifM gfp-apdA(P122A)-flag-lacZ Ω cat*	pSK88	PY79	This study
SKB72	*amyE::PmifM gfp-apdA(T111A)-flag-lacZ Ω cat*	pSK78	PY79	This study
SKB73	*amyE::PmifM gfp-apdA(A121S)-flag-lacZ Ω cat*	pSK87	PY79	This study
SKB74	*amyE::PmifM gfp-apdA(D119A)-flag-lacZ Ω cat*	pSK95	PY79	This study
SKB79	*amyE::PmifM gfp-apdP-flag-lacZd9 Ω cat*	pSK92	PY79	This study
SKB81	*amyE::PmifM gfp-apdP(Q126A)-flag-lacZd9 Ω cat*	pSK97	PY79	This study
SKB82	*amyE::PmifM gfp-apdP(S127A)-flag-lacZd9 Ω cat*	pSK109	PY79	This study
SKB84	*amyE::PmifM gfp-apdP(K128A)-flag-lacZd9 Ω cat*	pSK98	PY79	This study
SKB85	*amyE::PmifM gfp-apdP(C129A)-flag-lacZd9 Ω cat*	pSK99	PY79	This study
SKB86	*amyE::PmifM gfp-apdP(I130A)-flag-lacZd9 Ω cat*	pSK100	PY79	This study
SKB87	*amyE::PmifM gfp-apdP(R131A)-flag-lacZd9 Ω cat*	pSK107	PY79	This study
SKB88	*amyE::PmifM gfp-apdP(A132S)-flag-lacZd9 Ω cat*	pSK106	PY79	This study
SKB89	*amyE::PmifM gfp-apdP(P133A)-flag-lacZd9 Ω cat*	pSK101	PY79	This study
SKB258	*amyE::PmifM gfp-apcA(smt1)-flag-lacZ Ω cat*	pSK286	PY79	This study
SKB259	*amyE::PmifM gfp-apcA(smt2)-flag-lacZ Ω cat*	pSK287	PY79	This study
SKB260	*amyE::PmifM gfp-apcA(smt3)-flag-lacZ Ω cat*	pSK288	PY79	This study
SKB261	*amyE::PmifM gfp-apdA(smt1)-flag-lacZ Ω cat*	pSK289	PY79	This study
SKB262	*amyE::PmifM gfp-apdA(smt2)-flag-lacZ Ω cat*	pSK290	PY79	This study
SKB263	*amyE::PmifM gfp-apdA(smt3)-flag-lacZ Ω cat*	pSK291	PY79	This study
SKB264	*amyE::PmifM gfp-apdP(smt1)-flag-lacZ Ω cat*	pSK293	PY79	This study
SKB265	*amyE::PmifM gfp-apdP(smt2)-flag-lacZ Ω cat*	pSK294	PY79	This study
SKB266	*amyE::PmifM gfp-apdP(smt3)-flag-lacZ Ω cat*	pSK295	PY79	This study
SKB267	*amyE::PmifM gfp-apdP(smt4)-flag-lacZ Ω cat*	pSK296	PY79	This study
SKB296	*amyE::PmifM gfp-apcA-flag-lacZ(Δ Met) Ω cat*	pSK313	PY79	This study
SKB297	*amyE::PmifM gfp-apdA-flag-lacZ(Δ Met) Ω cat*	pSK314	PY79	This study
SKB298	*amyE::PmifM gfp-apdP-flag-lacZ(Δ Met) Ω cat*	pSK315	PY79	This study
SKB299	*amyE::PmifM gfp-apcA(R98A)-flag-lacZ(Δ Met) Ω cat*	pSK316	PY79	This study
SKB300	*amyE::PmifM gfp-apdA(R120A)-flag-lacZ(Δ Met) Ω cat*	pSK317	PY79	This study
SKB301	*amyE::PmifM gfp-apdP(R131A)-flag-lacZ(Δ Met) Ω cat*	pSK318	PY79	This study
SKB302	*amyE::PmifM gfp-GSGGSGG-apcA-flag-lacZ Ω cat*	pSK319	PY79	This study
SKB303	*amyE::PmifM gfp-GSGGSGG-apcA(R98A)-flag-lacZ Ω cat*	pSK320	PY79	This study
NAB1265	*amyE::PmifM gfp-apdA_GTA126GTT-flag-lacZ Ω cat*	pSK22	PY79	This study
NAB1267	*amyE::PmifM gfp-apdA_FS39-118-flag-lacZ Ω cat*	pSK27	PY79	This study
NAB1268	*amyE::PmifM gfp-apdA_FS39-108-flag-lacZ Ω cat*	pSK28	PY79	This study
NAB1269	*amyE::PmifM gfp-apdA_FS39-98-flag-lacZ Ω cat*	pSK29	PY79	This study
NAB1270	*amyE::PmifM gfp-apdA_FS39-128-flag-lacZ Ω cat*	pSK35	PY79	This study

ref

1) Youngman, P., Perkins, J. B. & Losick, R. Mol. Gen. Genet. 195, 424–433 (1984).

2) Chiba, S., Lamsa, A. & Pogliano, K. EMBO J. 28, 3461–3475 (2009).

Supplementary Table S3: Pladmids used in this study

Plasmid	Gene	Primer 1	Primer 2	Template	Primer 3	Primer 4	Template	ref
pCH746	*amyE::PmifM mifM-yidC2'-lacZ Ω cat*							1
pCH930	*amyE::PmifM gfp-mifM35-flag-yidC2'-lacZ Ω cat*							1
pCH2096	*amyE::PmifM apcA Ω cat*	SP1	SP2	Re_apcA (*1)	SP3	SP4	pCH746	This study
pCH2099	*amyE::PmifM apdA Ω cat*	SP1	SP2	Aj_apdA (*1)	SP3	SP4	pCH746	This study
pCH2102	*amyE::PmifM apdP Ω cat*	SP1	SP2	Sm_apdP (*1)	SP3	SP4	pCH746	This study
pCH2105	*amyE::PmifM gfp-apcA62-flag Ω cat*	SP5	SP6	pCH2096	SP7	SP8	pCH930	This study
pCH2107	*amyE::PmifM gfp-apdA39-flag Ω cat*	SP9	SP10	pCH2099	SP7	SP8	pCH930	This study
pCH2108	*amyE::PmifM gfp-apdP34-flag Ω cat*	SP11	SP12	pCH2102	SP7	SP8	pCH930	This study
pCH2118	*amyE::PmifM gfp-apcA62(R98A)-flag Ω cat*	SP13	SP14	pCH2105				This study
pCH2120	*amyE::PmifM gfp-apdA39(R120A)-flag Ω cat*	SP15	SP16	pCH2107				This study
pCH2122	*amyE::PmifM gfp-apdP34(R131A)-flag Ω cat*	SP17	SP18	pCH2108				This study
pCH2124	*amyE::PmifM gfp-apcA-flag-lacZ Ω cat*	SP19	SP20	pCH2105				This study
pCH2125	*amyE::PmifM gfp-apdA-flag-lacZ Ω cat*	SP19	SP20	pCH2107				This study
pCH2126	*amyE::PmifM gfp-apdP-flag-lacZ Ω cat*	SP19	SP20	pCH2108				This study
pCH2127	*amyE::PmifM gfp-apcA(R98A)-flag-lacZ Ω cat*	SP19	SP20	pCH2118				This study
pCH2128	*amyE::PmifM gfp-apdP(R131A)-flag-lacZ Ω cat*	SP19	SP20	pCH2122				This study
pCH2134	*amyE::PmifM gfp-apdA(R120A)-flag-lacZ Ω cat*	SP19	SP20	pCH2120				This study
pCH2140	*amyE::PmifM gfp-apcA62-yidC2'-lacZ Ω cat*	SP5	SP2	pCH2096	SP3	SP8	pCH930	This study
pCH2141	*amyE:PmifM gfp-apdP34-secDF2'-lacZ Ω cat*	SP11	SP2	pCH2102	SP3	SP8	pCH930	This study
pCH2143	*amyE::PmifM gfp-apdA39-secDF2'-lacZ Ω cat*	SP9	SP2	pCH2099	SP3	SP8	pCH930	This study
pCH2162	*amyE::PmifM gfp-apcA(G105A)-flag-lacZ Ω cat*	SP21	SP22	pCH2124				This study
pCH2163	*amyE::PmifM gfp-apcA(G105L)-flag-lacZ Ω cat*	SP21	SP22	pCH2124				This study
pCH2164	*amyE::PmifM gfp-apcA(G105R)-flag-lacZ Ω cat*	SP21	SP22	pCH2124				This study
pCH2166	*amyE::PmifM gfp-apcA(G105E)-flag-lacZ Ω cat*	SP21	SP22	pCH2124				This study
pCH2167	*amyE::PmifM gfp-apcA(G105W)-flag-lacZ Ω cat*	SP21	SP22	pCH2124				This study
pCH2168	*amyE::PmifM gfp-apcA(G105S)-flag-lacZ Ω cat*	SP21	SP22	pCH2124				This study
pCH2169	*amyE::PmifM gfp-apcA(G105Y)-flag-lacZ Ω cat*	SP21	SP22	pCH2124				This study
pCH2170	*amyE::PmifM gfp-apcA(G105P)-flag-lacZ Ω cat*	SP21	SP22	pCH2124				This study
pCH2171	*amyE::PmifM gfp-apcA(G105M)-flag-lacZ Ω cat*	SP21	SP22	pCH2124				This study
pCH2172	*amyE::PmifM gfp-apcA(G105V)-flag-lacZ Ω cat*	SP21	SP22	pCH2124				This study
pCH2173	*amyE::PmifM gfp-apcA(G105C)-flag-lacZ Ω cat*	SP21	SP22	pCH2124				This study
pCH2174	*amyE::PmifM gfp-apcA(G105D)-flag-lacZ Ω cat*	SP21	SP22	pCH2124				This study
pCH2175	*amyE::PmifM gfp-apdA(P123V)-flag-lacZ Ω cat*	SP23	SP24	pCH2125				This study
pCH2176	*amyE::PmifM gfp-apdA(P123A)-flag-lacZ Ω cat*	SP23	SP24	pCH2125				This study
pCH2177	*amyE::PmifM gfp-apdA(P123G)-flag-lacZ Ω cat*	SP23	SP24	pCH2125				This study
pCH2178	*amyE::PmifM gfp-apdA(P123N)-flag-lacZ Ω cat*	SP23	SP24	pCH2125				This study
pCH2179	*amyE::PmifM gfp-apdA(P123I)-flag-lacZ Ω cat*	SP23	SP24	pCH2125				This study
pCH2181	*amyE::PmifM gfp-apdA(P123S)-flag-lacZ Ω cat*	SP23	SP24	pCH2125				This study
pCH2183	*amyE::PmifM gfp-apdA(P123R)-flag-lacZ Ω cat*	SP23	SP24	pCH2125				This study
pCH2184	*amyE::PmifM gfp-apdA(P123F)-flag-lacZ Ω cat*	SP23	SP24	pCH2125				This study
pCH2185	*amyE::PmifM gfp-apdA(P123W)-flag-lacZ Ω cat*	SP23	SP24	pCH2125				This study
pCH2186	*amyE::PmifM gfp-apdA(P123K)-flag-lacZ Ω cat*	SP23	SP24	pCH2125				This study
pCH2187	*amyE::PmifM gfp-apdA(P123H)-flag-lacZ Ω cat*	SP23	SP24	pCH2125				This study
pCH2188	*amyE::PmifM gfp-apdA(P123C)-flag-lacZ Ω cat*	SP23	SP24	pCH2125				This study
pCH2189	*amyE::PmifM gfp-apdA(P123D)-flag-lacZ Ω cat*	SP23	SP24	pCH2125				This study
pCH2190	*amyE::PmifM gfp-apdA(P123L)-flag-lacZ Ω cat*	SP23	SP24	pCH2125				This study
pCH2191	*amyE::PmifM gfp-apdA(P123T)-flag-lacZ Ω cat*	SP23	SP24	pCH2125				This study
pCH2192	*amyE::PmifM gfp-apdA(P123M)-flag-lacZ Ω cat*	SP23	SP24	pCH2125				This study
pCH2194	*amyE::PmifM gfp-apdP(P134A)-flag-lacZ Ω cat*	SP25	SP26	pCH2126				This study
pCH2195	*amyE::PmifM gfp-apdP(P134Q)-flag-lacZ Ω cat*	SP25	SP26	pCH2126				This study
pCH2196	*amyE::PmifM gfp-apdP(P134T)-flag-lacZ Ω cat*	SP25	SP26	pCH2126				This study
pCH2197	*amyE::PmifM gfp-apdP(P134D)-flag-lacZ Ω cat*	SP25	SP26	pCH2126				This study
pCH2198	*amyE::PmifM gfp-apdP(P134L)-flag-lacZ Ω cat*	SP25	SP26	pCH2126				This study
pCH2199	*amyE::PmifM gfp-apdP(P134V)-flag-lacZ Ω cat*	SP25	SP26	pCH2126				This study
pCH2200	*amyE::PmifM gfp-apdP(P134F)-flag-lacZ Ω cat*	SP25	SP26	pCH2126				This study
pCH2201	*amyE::PmifM gfp-apdP(P134S)-flag-lacZ Ω cat*	SP25	SP26	pCH2126				This study
pCH2202	*amyE::PmifM gfp-apdP(P134G)-flag-lacZ Ω cat*	SP25	SP26	pCH2126				This study
pCH2203	*amyE::PmifM gfp-apdP(P134R)-flag-lacZ Ω cat*	SP25	SP26	pCH2126				This study
pCH2204	*amyE::PmifM gfp-apdP(P134W)-flag-lacZ Ω cat*	SP25	SP26	pCH2126				This study
pCH2205	*amyE::PmifM gfp-apdP(P134Y)-flag-lacZ Ω cat*	SP25	SP26	pCH2126				This study
pCH2206	*amyE::PmifM gfp-apcA(G105F)-flag-lacZ Ω cat*	SP27	SP22	pCH2124				This study
pCH2207	*amyE::PmifM gfp-apcA(G105T)-flag-lacZ Ω cat*	SP28	SP22	pCH2124				This study
pCH2208	*amyE::PmifM gfp-apcA(G105N)-flag-lacZ Ω cat*	SP29	SP22	pCH2124				This study
pCH2209	*amyE::PmifM gfp-apcA(G105K)-flag-lacZ Ω cat*	SP29	SP22	pCH2124				This study
pCH2210	*amyE::PmifM gfp-apcA(G105H)-flag-lacZ Ω cat*	SP29	SP22	pCH2124				This study
pCH2211	*amyE::PmifM gfp-apcA(G105I)-flag-lacZ Ω cat*	SP30	SP22	pCH2124				This study

pCH2212	*amyE::PmifM gfp-apcA(G105Q)-flag-lacZ Ω cat*	SP31	SP22	pCH2124				This study
pCH2213	*amyE::PmifM gfp-apdA(P123Y)-flag-lacZ Ω cat*	SP32	SP24	pCH2125				This study
pCH2214	*amyE::PmifM gfp-apdA(P123Q)-flag-lacZ Ω cat*	SP33	SP24	pCH2125				This study
pCH2215	*amyE::PmifM gfp-apdA(P123E)-flag-lacZ Ω cat*	SP34	SP24	pCH2125				This study
pCH2216	*amyE::PmifM gfp-apdP(P134I)-flag-lacZ Ω cat*	SP35	SP26	pCH2126				This study
pCH2217	*amyE::PmifM gfp-apdP(P134K)-flag-lacZ Ω cat*	SP36	SP26	pCH2126				This study
pCH2218	*amyE::PmifM gfp-apdP(P134N)-flag-lacZ Ω cat*	SP36	SP26	pCH2126				This study
pCH2219	*amyE::PmifM gfp-apdP(P134C)-flag-lacZ Ω cat*	SP36	SP26	pCH2126				This study
pCH2220	*amyE::PmifM gfp-apdP(P134M)-flag-lacZ Ω cat*	SP37	SP26	pCH2126				This study
pCH2221	*amyE::PmifM gfp-apdP(P134H)-flag-lacZ Ω cat*	SP38	SP26	pCH2126				This study
pCH2222	*amyE::PmifM gfp-apdP(P134E)-flag-lacZ Ω cat*	SP39	SP26	pCH2126				This study
pSK6	*amyE::PmifM gfp-apcA_FS62-108-flag-lacZ Ω cat*	SP40	SP41	pCH2124	SP42	SP43	pCH2124	This study
pSK8	*amyE::PmifM gfp-apcA_FS62-98-flag-lacZ Ω cat*	SP40	SP44	pCH2124	SP42	SP45	pCH2124	This study
pSK9	*amyE::PmifM gfp-apcA_FS62-88-flag-lacZ Ω cat*	SP40	SP46	pCH2124	SP42	SP47	pCH2124	This study
pSK13	*amyE::PmifM gfp-apcA_FS62-78-flag-lacZ Ω cat*	SP40	SP48	pCH2124	SP42	SP49	pCH2124	This study
pSK14	*amyE::PmifM gfp-apdP_FS34-140-flag-lacZ Ω cat*	SP50	SP51	pCH2126	SP52	SP53	pCH2126	This study
pSK16	*amyE::PmifM gfp-apdP_FS34-130-flag-lacZ Ω cat*	SP50	SP54	pCH2126	SP55	SP53	pCH2126	This study
pSK18	*amyE::PmifM gfp-apdP_FS34-120-flag-lacZ Ω cat*	SP50	SP56	pCH2126	SP57	SP53	pCH2126	This study
pSK20	*amyE::PmifM gfp-apdP_FS34-110-flag-lacZ Ω cat*	SP50	SP58	pCH2126	SP59	SP53	pCH2126	This study
pSK22	*amyE::PmifM gfp-apdA_GTA126GTT-flag-lacZ Ω cat*	SP60	SP61	pCH2125	SP62	SP63	pCH2125	This study
pSK24	*amyE::PmifM gfp-apdP_FS34-125-flag-lacZ Ω cat*	SP50	SP64	pCH2126	SP65	SP53	pCH2126	This study
pSK27	*amyE::PmifM gfp-apdA_FS39-118-flag-lacZ Ω cat*	SP66	SP67	pSK22	SP68	SP69	pSK22	This study
pSK28	*amyE::PmifM gfp-apdA_FS39-108-flag-lacZ Ω cat*	SP66	SP70	pSK22	SP68	SP71	pSK22	This study
pSK29	*amyE::PmifM gfp-apdA_FS39-98-flag-lacZ Ω cat*	SP66	SP72	pSK22	SP68	SP73	pSK22	This study
pSK30	*amyE::PmifM gfp-apcA_FS62-73-flag-lacZ Ω cat*	SP40	SP74	pCH2124	SP42	SP75	pCH2124	This study
pSK31	*amyE::PmifM gfp-apdA_FS39-flag-lacZ Ω cat*	SP66	SP62	pSK22	SP69	SP60	pSK22	This study
pSK35	*amyE::PmifM gfp-apdA_FS39-128-flag-lacZ Ω cat*	SP76	SP62	pSK31	SP77	SP60	pSK31	This study
pSK38	*amyE::PmifM gfp-apcA(T74A)-flag-lacZ Ω cat*	SP78	SP62	pCH2124	SP79	SP60	pCH2124	This study
pSK39	*amyE::PmifM gfp-apcA(G75A)-flag-lacZ Ω cat*	SP80	SP62	pCH2124	SP81	SP60	pCH2124	This study
pSK40	*amyE::PmifM gfp-apcA(P76A)-flag-lacZ Ω cat*	SP82	SP62	pCH2124	SP83	SP60	pCH2124	This study
pSK41	*amyE::PmifM gfp-apcA(S77A)-flag-lacZ Ω cat*	SP84	SP62	pCH2124	SP85	SP60	pCH2124	This study
pSK42	*amyE::PmifM gfp-apcA(D78A)-flag-lacZ Ω cat*	SP86	SP62	pCH2124	SP87	SP60	pCH2124	This study
pSK43	*amyE::PmifM gfp-apcA(D79A)-flag-lacZ Ω cat*	SP88	SP62	pCH2124	SP89	SP60	pCH2124	This study
pSK44	*amyE::PmifM gfp-apcA(E80A)-flag-lacZ Ω cat*	SP90	SP62	pCH2124	SP91	SP60	pCH2124	This study
pSK45	*amyE::PmifM gfp-apcA(R81A)-flag-lacZ Ω cat*	SP92	SP62	pCH2124	SP93	SP60	pCH2124	This study
pSK46	*amyE::PmifM gfp-apcA(C82A)-flag-lacZ Ω cat*	SP94	SP62	pCH2124	SP95	SP60	pCH2124	This study
pSK47	*amyE::PmifM gfp-apcA(L83A)-flag-lacZ Ω cat*	SP96	SP62	pCH2124	SP97	SP60	pCH2124	This study
pSK48	*amyE::PmifM gfp-apcA(R84A)-flag-lacZ Ω cat*	SP98	SP62	pCH2124	SP99	SP60	pCH2124	This study
pSK49	*amyE::PmifM gfp-apcA(G85A)-flag-lacZ Ω cat*	SP100	SP62	pCH2124	SP101	SP60	pCH2124	This study
pSK50	*amyE::PmifM gfp-apcA(A86S)-flag-lacZ Ω cat*	SP102	SP62	pCH2124	SP103	SP60	pCH2124	This study
pSK51	*amyE::PmifM gfp-apcA(F87A)-flag-lacZ Ω cat*	SP104	SP62	pCH2124	SP105	SP60	pCH2124	This study
pSK52	*amyE::PmifM gfp-apcA(R88A)-flag-lacZ Ω cat*	SP106	SP62	pCH2124	SP107	SP60	pCH2124	This study
pSK53	*amyE::PmifM gfp-apcA(R89A)-flag-lacZ Ω cat*	SP108	SP62	pCH2124	SP109	SP60	pCH2124	This study
pSK54	*amyE::PmifM gfp-apcA(Q90A)-flag-lacZ Ω cat*	SP110	SP62	pCH2124	SP111	SP60	pCH2124	This study
pSK55	*amyE::PmifM gfp-apcA(S91A)-flag-lacZ Ω cat*	SP112	SP62	pCH2124	SP113	SP60	pCH2124	This study
pSK56	*amyE::PmifM gfp-apcA(S92A)-flag-lacZ Ω cat*	SP114	SP62	pCH2124	SP115	SP60	pCH2124	This study
pSK57	*amyE::PmifM gfp-apcA(A95S)-flag-lacZ Ω cat*	SP116	SP62	pCH2124	SP117	SP60	pCH2124	This study
pSK58	*amyE::PmifM gfp-apcA(P93A)-flag-lacZ Ω cat*	SP118	SP62	pCH2124	SP119	SP60	pCH2124	This study
pSK59	*amyE::PmifM gfp-apcA(D94A)-flag-lacZ Ω cat*	SP120	SP62	pCH2124	SP121	SP60	pCH2124	This study
pSK60	*amyE::PmifM gfp-apcA(G97A)-flag-lacZ Ω cat*	SP122	SP62	pCH2124	SP123	SP60	pCH2124	This study
pSK61	*amyE::PmifM gfp-apcA(P99A)-flag-lacZ Ω cat*	SP124	SP62	pCH2124	SP125	SP60	pCH2124	This study
pSK62	*amyE::PmifM gfp-apcA(P101A)-flag-lacZ Ω cat*	SP126	SP62	pCH2124	SP127	SP60	pCH2124	This study
pSK63	*amyE::PmifM gfp-apcA(R102A)-flag-lacZ Ω cat*	SP128	SP62	pCH2124	SP129	SP60	pCH2124	This study
pSK64	*amyE::PmifM gfp-apcA(A103S)-flag-lacZ Ω cat*	SP130	SP62	pCH2124	SP131	SP60	pCH2124	This study
pSK65	*amyE::PmifM gfp-apcA(Q100A)-flag-lacZ Ω cat*	SP132	SP62	pCH2124	SP133	SP60	pCH2124	This study
pSK66	*amyE::PmifM gfp-apcA(P104A)-flag-lacZ Ω cat*	SP134	SP62	pCH2124	SP135	SP60	pCH2124	This study
pSK67	*amyE::PmifM gfp-apcA(P96A)-flag-lacZ Ω cat*	SP136	SP62	pCH2124	SP137	SP60	pCH2124	This study
pSK76	*amyE::PmifM gfp-apdA(S109A)-flag-lacZ Ω cat*	SP138	SP62	pCH2125	SP139	SP60	pCH2125	This study
pSK77	*amyE::PmifM gfp-apdA(R110A)-flag-lacZ Ω cat*	SP140	SP62	pCH2125	SP141	SP60	pCH2125	This study
pSK78	*amyE::PmifM gfp-apdA(T111A)-flag-lacZ Ω cat*	SP142	SP62	pCH2125	SP143	SP60	pCH2125	This study
pSK79	*amyE::PmifM gfp-apdA(A112S)-flag-lacZ Ω cat*	SP144	SP62	pCH2125	SP145	SP60	pCH2125	This study
pSK80	*amyE::PmifM gfp-apdA(N113A)-flag-lacZ Ω cat*	SP146	SP62	pCH2125	SP147	SP60	pCH2125	This study
pSK81	*amyE::PmifM gfp-apdA(R114A)-flag-lacZ Ω cat*	SP148	SP62	pCH2125	SP149	SP60	pCH2125	This study
pSK82	*amyE::PmifM gfp-apdA(L115A)-flag-lacZ Ω cat*	SP150	SP62	pCH2125	SP151	SP60	pCH2125	This study
pSK83	*amyE::PmifM gfp-apdA(A116S)-flag-lacZ Ω cat*	SP152	SP62	pCH2125	SP153	SP60	pCH2125	This study
pSK84	*amyE::PmifM gfp-apdA(L117A)-flag-lacZ Ω cat*	SP154	SP62	pCH2125	SP155	SP60	pCH2125	This study
pSK85	*amyE::PmifM gfp-apdA(G118A)-flag-lacZ Ω cat*	SP156	SP62	pCH2125	SP157	SP60	pCH2125	This study
pSK87	*amyE::PmifM gfp-apdA(A121S)-flag-lacZ Ω cat*	SP158	SP62	pCH2125	SP159	SP60	pCH2125	This study

pSK88	*amyE::PmifM gfp-apdA(P122A)-flag-lacZ Ω cat*	SP160	SP62	pCH2125	SP161	SP60	pCH2125	This study
pSK92	*amyE::PmifM gfp-apdP-flag-lacZd9 Ω cat*	SP162	SP62	pSK69	SP163	SP60	pSK69	This study
pSK95	*amyE::PmifM gfp-apdA(D119A)-flag-lacZ Ω cat*	SP164	SP62	pCH2125	SP165	SP60	pCH2125	This study
pSK97	*amyE::PmifM gfp-apdP(Q126A)-flag-lacZd9 Ω cat*	SP166	SP62	pSK92	SP167	SP60	pSK92	This study
pSK98	*amyE::PmifM gfp-apdP(K128A)-flag-lacZd9 Ω cat*	SP168	SP62	pSK92	SP169	SP60	pSK92	This study
pSK99	*amyE::PmifM gfp-apdP(C129A)-flag-lacZd9 Ω cat*	SP170	SP62	pSK92	SP171	SP60	pSK92	This study
pSK100	*amyE::PmifM gfp-apdP(I130A)-flag-lacZd9 Ω cat*	SP172	SP62	pSK92	SP173	SP60	pSK92	This study
pSK101	*amyE::PmifM gfp-apdP(P133A)-flag-lacZd9 Ω cat*	SP174	SP62	pSK92	SP175	SP60	pSK92	This study
pSK106	*amyE::PmifM gfp-apdP(A132S)-flag-lacZd9 Ω cat*	SP176	SP62	pSK92	SP177	SP60	pSK92	This study
pSK107	*amyE::PmifM gfp-apdP(R131A)-flag-lacZd9 Ω cat*	SP178	SP62	pSK92	SP179	SP60	pSK92	This study
pSK109	*amyE::PmifM gfp-apdP(S127A)-flag-lacZd9 Ω cat*	SP180	SP181	pSK92	SP182	SP183	pSK92	This study
pSK286	*amyE::PmifM gfp-apcA(smt1)-flag-lacZ Ω cat*	SP184	SP129	pCH2124				This study
pSK287	*amyE::PmifM gfp-apcA(smt2)-flag-lacZ Ω cat*	SP184	SP129	pCH2124				This study
pSK288	*amyE::PmifM gfp-apcA(smt3)-flag-lacZ Ω cat*	SP184	SP129	pCH2124				This study
pSK289	*amyE::PmifM gfp-apdA(smt1)-flag-lacZ Ω cat*	SP185	SP186	pCH2125				This study
pSK290	*amyE::PmifM gfp-apdA(smt2)-flag-lacZ Ω cat*	SP185	SP186	pCH2125				This study
pSK291	*amyE::PmifM gfp-apdA(smt3)-flag-lacZ Ω cat*	SP185	SP186	pCH2125				This study
pSK293	*amyE::PmifM gfp-apdP(smt1)-flag-lacZ Ω cat*	SP187	SP188	pCH2126				This study
pSK294	*amyE::PmifM gfp-apdP(smt2)-flag-lacZ Ω cat*	SP187	SP188	pCH2126				This study
pSK295	*amyE::PmifM gfp-apdP(smt3)-flag-lacZ Ω cat*	SP187	SP188	pCH2126				This study
pSK296	*amyE::PmifM gfp-apdP(smt4)-flag-lacZ Ω cat*	SP187	SP188	pCH2126				This study
pSK313	*amyE::PmifM gfp-apcA-flag-lacZ(DMet) Ω cat*	SP189	SP062	pCH2124	SP190	SP060	pCH2124	This study
pSK314	*amyE::PmifM gfp-apdA-flag-lacZ(DMet) Ω cat*	SP189	SP062	pCH2125	SP190	SP060	pCH2125	This study
pSK315	*amyE::PmifM gfp-apdP-flag-lacZ(DMet) Ω cat*	SP189	SP062	pCH2126	SP190	SP060	pCH2126	This study
pSK316	*amyE::PmifM gfp-apcA(R98A)-flag-lacZ(DMet) Ω cat*	SP189	SP062	pCH2127	SP190	SP060	pCH2127	This study
pSK317	*amyE::PmifM gfp-apdA(R120A)-flag-lacZ(DMet) Ω cat*	SP189	SP062	pCH2134	SP190	SP060	pCH2134	This study
pSK318	*amyE::PmifM gfp-apdP(R131A)-flag-lacZ(DMet) Ω cat*	SP189	SP062	pCH2128	SP190	SP060	pCH2128	This study
pSK319	*amyE::PmifM gfp-GSGGSGG-apcA-flag-lacZ Ω cat*	SP191	SP181	pCH2124	SP192	SP183	pCH2124	This study
pSK320	*amyE::PmifM gfp-GSGGSGG-apcA(R98A)-flag-lacZ Ω cat*	SP191	SP181	pCH2127	SP192	SP183	pCH2127	This study

(*1) Synthetic oligonucleotide

ref

1) Chiba, S., Lamsa, A. & Pogliano, K. EMBO J. 28, 3461–3475 (2009).

Supplementary Table S4: Primers used in this study

Name	Sequence
SP001	ATAGTAAAATGAAGCTATTGA
SP002	ATCCGTAATCATGGTACTAGT
SP003	ACTAGTACCATGATTACGGAT
SP004	TCAATAGCTTCATTTTACTATA
SP005	GGCATGGATGAACTATACAAAAAGTACGAACTTCTGCAGCTG
SP006	GTCGTCGTCGTCTTTATAGTCCAGACCGATTCCCGGAGCTCG
SP007	GACTATAAAGACGACGACGAC
SP008	GGCATGGATGAACTATACAAA
SP009	GGCATGGATGAACTATACAAAGACGAGTCGCGCGGGGCGAAC
SP010	GTCGTCGTCGTCTTTATAGTCGACGGCTACCGGGAAAGGAGG
SP011	GGCATGGATGAACTATACAAAATCGGCCAGAGTGCCGCGTCC
SP012	GTCGTCGTCGTCTTTATAGTCAAGGAAGGCTCCCGCCGCTGG
SP013	GCGCCCGGCGCTCCACAGCCTCGAGCT
SP014	CTGTGGAGCGCCGGGCGCGTCCGGGCT
SP015	CTGGGTGATGCCGCTCCTCCT
SP016	AGCGGCATCACCCAGCGCGAGGCGGTT
SP017	AAGTGCATTGCCGCGCCGCCA
SP018	CGCGGCAATGCACTTGCTCTGACAGGA
SP019	TATAAAGACGACGACGACAAATTAAAAACATATCAAACTAGT
SP020	TTTGTCGTCGTCGTCTTTATA
SP021	CCACAGCCTCGAGCTCCGNNNATCGGTCTGGACTATAAAGAC
SP022	CGGAGCTCGAGGCTGTGGACG
SP023	CTGGGTGATCGCGCTCCTNNNTTCCCGGTAGCCGTCGACTAT
SP024	AGGAGCGCGATCACCCAGCGC
SP025	AAGTGCATTCGCGCGCCGNNNGCGGCGGGAGCCTTCCTTGAC
SP026	CGGCGCGCGAATGCACTTGCT
SP027	CCACAGCCTCGAGCTCCGWTTATCGGTCTGGACTATAAAGAC
SP028	CCACAGCCTCGAGCTCCGACTATCGGTCTGGACTATAAAGAC
SP029	CCACAGCCTCGAGCTCCGMAWATCGGTCTGGACTATAAAGAC
SP030	CCACAGCCTCGAGCTCCGATTATCGGTCTGGACTATAAAGAC
SP031	CCACAGCCTCGAGCTCCGCAGATCGGTCTGGACTATAAAGAC
SP032	CTGGGTGATCGCGCTCCTBAWTTCCCGGTAGCCGTCGACTAT
SP033	CTGGGTGATCGCGCTCCTCAGTTCCCGGTAGCCGTCGACTAT
SP034	CTGGGTGATCGCGCTCCTGAATTCCCGGTAGCCGTCGACTAT
SP035	AAGTGCATTCGCGCGCCGATKGCGGCGGGAGCCTTCCTTGAC
SP036	AAGTGCATTCGCGCGCCGVAWGCGGCGGGAGCCTTCCTTGAC
SP037	AAGTGCATTCGCGCGCCGATGGCGGCGGGAGCCTTCCTTGAC
SP038	AAGTGCATTCGCGCGCCGCATGCGGCGGGAGCCTTCCTTGAC
SP039	AAGTGCATTCGCGCGCCGGAAGCGGCGGGAGCCTTCCTTGAC
SP040	GGCATGGATGAACTATACAAAAGTACGAACTTCTGCAGCTGT
SP041	GTCGTCGTCGTCTTTATAGTCACAGACCGATTCCCGGAGCTCG
SP042	GACTATAAAGACGACGACGAC
SP043	TTTGTATAGTTCATCCATGCC
SP044	TCCCGGAGCTCGAGGCTGTGGAACGGCCGGGCGCGTCCGGGCT
SP045	CCACAGCCTCGAGCTCCGGGA
SP046	CGCGTCCGGGCTGCTCTGCCTAGCGGAAAGCGCCACGCAGGCA
SP047	AGGCAGAGCAGCCCGGACGCG
SP048	GCCACGCAGGCAGCGTTCGTCAATCAGAGGGGCCAGTGGCCGA
SP049	GACGAACGCTGCCTGCGTGGC
SP050	GGCATGGATGAACTATACAAAAATCGGCCAGAGTGCCGCGTCC
SP051	GTCGTCGTCGTCTTTATAGTCAGGAAGGCTCCCGCCGCTGGC
SP052	GACTATAAAGACGACGACGAC
SP053	TTTGTATAGTTCATCCATGCC
SP054	TCCCGCCGCTGGCGGCGCGCGATGCACTTGCTCTGACAGGAT
SP055	CGCGCGCCGCCAGCGGCGGGA
SP056	GCTCTGACAGGATGGGAGACGGCAATTCTGGTTTTCGCCACC
SP057	CGTCTCCCATCCTGTCAGAGC
SP058	GGTTTTCGCCACCGGCAGGGAGGAGCCATCTCTGGCGCAATC
SP059	TCCCTGCCGGTGGCGAAAACC
SP060	GGTATGATTGATGACCGCGT
SP061	ACCGGGAAAGGAGGAGCGCGA
SP062	ACGCGGTCATCAATCATACC
SP063	TCGCGCTCCTCCTTTCCCGGTTGCCGTCGACTATAAAGACGAC
SP064	CGCGCGAATGCACTTGCTCTGCAGGATGGGAGACGCGCAATT
SP065	CAGAGCAAGTGCATTCGCGCG
SP066	ATGGATGAACTATACAAAGACAGTCGCGCGGGGCGAACGCGAA
SP067	CGGGAAAGGAGGAGCGCGATCAACCCAGCGCGAGGCGGTTCGC
SP068	TCGCGCACGGCGAACCGCCTT
SP069	GTCTTTGTATAGTTCATCCAT
SP070	GAGGCGGTTCGCCGTGCGCGAAGGCGGGAGCTGCTTCGTCGTG
SP071	TCGCGCACGGCGAACCGCCTC
SP072	TGCTTCGTCGTGGAGCTCGCTAGAGGCGGAGCAGCGTCGTGGA
SP073	AGCGAGCTCCACGACGAAGCA
SP074	TTCGTCATCAGAGGGGCCAGTAGGCCGAAACCGCGCACAGCTG
SP075	ACTGGCCCCTCTGATGACGAA
SP076	GTCGTCGTCGTCTTTATAGTCAGACGGCAACCGGGAAAGGAGG
SP077	GACTATAAAGACGACGACGAC
SP078	CAGCTGTGCGCGGTTTCGGCCGCTGGCCCCTCTGATGACGAACGC
SP079	GGCCGAAACCGCGCACAGCTG
SP080	CTGTGCGCGGTTTCGGCCACTGCCCCCTCTGATGACGAACGCTGC
SP081	AGTGGCCGAAACCGCGCACAG
SP082	TGCGCGGTTTCGGCCACTGGCGCCTCTGATGACGAACGCTGCCTG
SP083	GCCAGTGGCCGAAACCGCGCA
SP084	GCGGTTTCGGCCACTGGCCCCGCTGATGACGAACGCTGCCTGCGT
SP085	GGGGCCAGTGGCCGAAACCGC
SP086	GTTTCGGCCACTGGCCCCTCTGCTGACGAACGCTGCCTGCGTGGC
SP087	AGAGGGGCCAGTGGCCGAAAC
SP088	TCGGCCACTGGCCCCTCTGATGCCGAACGCTGCCTGCGTGGCGCT
SP089	ATCAGAGGGGCCAGTGGCCGA
SP090	GCCACTGGCCCCTCTGATGACGCACGCTGCCTGCGTGGCGCTTTC
SP091	GTCATCAGAGGGGCCAGTGGC
SP092	ACTGGCCCCTCTGATGACGAAGCCTGCCTGCGTGGCGCTTTCCGC
SP093	TTCGTCATCAGAGGGGCCAGT
SP094	GGCCCCTCTGATGACGAACGCGCCCTGCGTGGCGCTTTCCGCAGG
SP095	GCGTTCGTCATCAGAGGGGCC
SP096	CCCTCTGATGACGAACGCTGCGCGCGTGGCGCTTTCCGCAGGCAG
SP097	GCAGCGTTCGTCATCAGAGGG
SP098	TCTGATGACGAACGCTGCCTGGCTGGCGCTTTCCGCAGGCAGAGC
SP099	CAGGCAGCGTTCGTCATCAGA
SP100	GATGACGAACGCTGCCTGCGTGCCGCTTTCCGCAGGCAGAGCAGC
SP101	ACGCAGGCAGCGTTCGTCATC
SP102	GACGAACGCTGCCTGCGTGGCTCTTTCCGCAGGCAGAGCAGCCCG
SP103	GCCACGCAGGCAGCGTTCGTC
SP104	GAACGCTGCCTGCGTGGCGCTGCCCGCAGGCAGAGCAGCCCGGAC

SP105	AGCGCCACGCAGGCAGCGTTC
SP106	CGCTGCCTGCGTGGCGCTTTCGCCAGGCAGAGCAGCCCGGACGCG
SP107	GAAAGCGCCACGCAGGCAGCG
SP108	TGCCTGCGTGGCGCTTTCCGCGCGCAGAGCAGCCCGGACGCGCCC
SP109	GCGGAAAGCGCCACGCAGGCA
SP110	CTGCGTGGCGCTTTCCGCAGGGCGAGCAGCCCGGACGCGCCCGGC
SP111	CCTGCGGAAAGCGCCACGCAG
SP112	CGTGGCGCTTTCCGCAGGCAGGCCAGCCCGGACGCGCCCGGCCGT
SP113	CTGCCTGCGGAAAGCGCCACG
SP114	GGCGCTTTCCGCAGGCAGAGCGCCCCGGACGCGCCCGGCCGTCCA
SP115	GCTCTGCCTGCGGAAAGCGCC
SP116	CGCAGGCAGAGCAGCCCGGACTCGCCCGGCCGTCCACAGCCTCGA
SP117	GTCCGGGCTGCTCTGCCTGCG
SP118	GCTTTCCGCAGGCAGAGCAGCGCGGACGCGCCCGGCCGTCCACAG
SP119	GCTGCTCTGCCTGCGGAAAGC
SP120	TTCCGCAGGCAGAGCAGCCCGGCCGCGCCCGGCCGTCCACAGCCT
SP121	CGGGCTGCTCTGCCTGCGGAA
SP122	CAGAGCAGCCCGGACGCGCCCGCCCGTCCACAGCCTCGAGCTCCG
SP123	GGGCGCGTCCGGGCTGCTCTG
SP124	AGCCCGGACGCGCCCGGCCGTGCACAGCCTCGAGCTCCGGGAATC
SP125	ACGGCCGGGCGCGTCCGGGCT
SP126	GACGCGCCCGGCCGTCCACAGGCTCGAGCTCCGGGAATCGGTCTG
SP127	CTGTGGACGGCCGGGCGCGTC
SP128	GCGCCCGGCCGTCCACAGCCTGCAGCTCCGGGAATCGGTCTGGAC
SP129	AGGCTGTGGACGGCCGGGCGC
SP130	CCCGGCCGTCCACAGCCTCGATCTCCGGGAATCGGTCTGGACTAT
SP131	TCGAGGCTGTGGACGGCCGGG
SP132	CCGGACGCGCCCGGCCGTCCAGCGCCTCGAGCTCCGGGAATCGGT
SP133	TGGACGGCCGGGCGCGTCCGG
SP134	GGCCGTCCACAGCCTCGAGCTGCGGGAATCGGTCTGGACTATAAA
SP135	AGCTCGAGGCTGTGGACGGCC
SP136	AGGCAGAGCAGCCCGGACGCGGCCGGCCGTCCACAGCCTCGAGCT
SP137	CGCGTCCGGGCTGCTCTGCCT
SP138	CACGACGAAGCAGCTCCCGCCGCGCGCACGGCGAACCGCCTCGCG
SP139	GGCGGGAGCTGCTTCGTCGTG
SP140	GACGAAGCAGCTCCCGCCTCGGCCACGGCGAACCGCCTCGCGCTG
SP141	CGAGGCGGGAGCTGCTTCGTC
SP142	GAAGCAGCTCCCGCCTCGCGCGCGGCGAACCGCCTCGCGCTGGGT
SP143	GCGCGAGGCGGGAGCTGCTTCA
SP144	GCAGCTCCCGCCTCGCGCACGTCGAACCGCCTCGCGCTGGGTGAT
SP145	CGTGCGCGAGGCGGGAGCTGC
SP146	GCTCCCGCCTCGCGCACGGCGGCCCGCCTCGCGCTGGGTGATCGC
SP147	CGCCGTGCGCGAGGCGGGAGC
SP148	CCCGCCTCGCGCACGGCGAACGCCCTCGCGCTGGGTGATCGCGCT
SP149	GTTCGCCGTGCGCGAGGCGGG
SP150	GCCTCGCGCACGGCGAACCGCGCCGCGCTGGGTGATCGCGCTCCT
SP151	GCGGTTCGCCGTGCGCGAGGC
SP152	TCGCGCACGGCGAACCGCCTCTCGCTGGGTGATCGCGCTCCTCCT
SP153	GAGGCGGTTCGCCGTGCGCGA
SP154	CGCACGGCGAACCGCCTCGCGGCGGGTGATCGCGCTCCTCCTTTC
SP155	CGCGAGGCGGTTCGCCGTGCG
SP156	ACGGCGAACCGCCTCGCGCTGGCTGATCGCGCTCCTCCTTTCCCG
SP157	CAGCGCGAGGCGGTTCGCCGT
SP158	CGCCTCGCGCTGGGTGATCGCTCTCCTCCTTTCCCGGTAGCCGTC
SP159	GCGATCACCCAGCGCGAGGCG
SP160	CTCGCGCTGGGTGATCGCGCTGCTCCTTTCCCGGTAGCCGTCGAC
SP161	AGCGCGATCACCCAGCGCGAG
SP162	TTAAAAACATATCAAACTAGTGTCGTTTTACAACGTCGTGAC
SP163	ACTAGTTTGATATGTTTTTAA
SP164	GCGAACCGCCTCGCGCTGGGTGCTCGCGCTCCTCCTTTCCCGGTA
SP165	ACCCAGCGCGAGGCGGTTCGC
SP166	ATTGCGCGTCTCCCATCCTGTGCGAGCAAGTGCATTCGCGCGCCG
SP167	ACAGGATGGGAGACGCGCAAT
SP168	CGTCTCCCATCCTGTCAGAGCGCGTGCATTCGCGCGCCGCCAGCG
SP169	GCTCTGACAGGATGGGAGACG
SP170	CTCCCATCCTGTCAGAGCAAGGCCATTCGCGCGCCGCCAGCGGCG
SP171	CTTGCTCTGACAGGATGGGAG
SP172	CCATCCTGTCAGAGCAAGTGCGCTCGCGCGCCGCCAGCGGCGGGAA
SP173	GCACTTGCTCTGACAGGATGG
SP174	CAGAGCAAGTGCATTCGCGCGGCGCCAGCGGCGGGAGCCTTCCTT
SP175	CGCGCGAATGCACTTGCTCTG
SP176	TGTCAGAGCAAGTGCATTCGCTCGCCGCCAGCGGCGGGAGCCTTC
SP177	GCGAATGCACTTGCTCTGACA
SP178	AAGTGCATTGCCGCGCCGCCA
SP179	CGCGGCAATGCACTTGCTCTGACAGGA
SP180	GCGCGTCTCCCATCCTGTCAGGCCAAGTGCATTCGCGCGCCGCCA
SP181	CCCTTTCGTCTTCAAGAATT
SP182	CTGACAGGATGGGAGACGCGC
SP183	AATTCTTGAAGACGAAAGGG
SP184	GGCCGTCCACAGCCTAGGGCVCCHGGBATCGGTCTGGACTATAAA
SP185	CTCGCGCTGGGTGATAGRGCVCCVCCVTTCCCGGTAGCCGTCGAC
SP186	ATCACCCAGCGCGAGGCGGTT
SP187	CAGAGCAAGTGCATTAGRGCHCCHCCBGCGGCGGGAGCCTTCCTT
SP188	AATGCACTTGCTCTGACAGGA
SP189	TGAAGCTAGGAGGAGGATGTGTACACATACTTTGTGGGATCCAAGCTTACT
SP190	CACATCCTCCTCCTAGCTTCA
SP191	GGTAGTGGTGGTAGTGGTGGTAAGTACGAACTTCTGCAGCTG
SP192	ACCACCACTACCACCACTACCTTTGTATAGTTCATCCATGCC
Primers for in vitro translation	
PP1	TAACTTTAAGAAGGAGATATACCAATGATGACAATGTTTGTGGGATC
PP2	AATTCAGACGGCAAACGACTG
PP3	GAAATTAATACGACTCACTATAGGGAGACCACAACGGTTTCCCTCTAGAAATAATTTTGTTTAACTTTAAGAAGGAG
PP4	TAACTTTAAGAAGGAGGGAGATATACCAATGACAATGTTTGTGGGATC
PP5	TACGCCAGCTGGCGAAAGGG
PP6	CGGAGCTCGAGGCTGTGGACG
PP7	TCCAGCTCGAGGCTGTGGACG
PP8	TCAGACGGCTACCGGGAAGGGTGGAGCGCGATCACCCAGCGC
PP9	TCAGACGGCTACCGGGAATGGGGGAGCGCGATCACCCAGCGC
PP10	TGGAGCGCGATCACCCAGCGCGAG
PP11	GGGAGCGCGATCACCCAGCGCGAG
PP12	TCAAAGGAAGGCTCCCGCCGCGGGTGGCGCGCGAATGCA
PP13	TCAAAGGAAGGCTCCCGCCGCTGGGGGCGCGCGAATGCA
PP14	TGGCGCGCGAATGCACTTGCT
PP15	GGGCGCGCGAATGCACTTGCT

Supplementary Table S5: *E. coli* strains used in this study

Strains	Genotype
JM109 / pCH2105	*amyE::PmifM gfp-apcA62-flag Ω cat*
JM109 / pCH2107	*amyE::PmifM gfp-apdA39-flag Ω cat*
JM109 / pCH2108	*amyE::PmifM gfp-apdP34-flag Ω cat*
JM109 / pCH2118	*amyE::PmifM gfp-apcA62(R98A)-flag Ω cat*
JM109 / pCH2120	*amyE::PmifM gfp-apdA39(R120A)-flag Ω cat*
JM109 / pCH2122	*amyE::PmifM gfp-apdP34(R131A)-flag Ω cat*
JM109 / pSK97	*amyE::PmifM gfp-apdP(Q126A)-flag-lacZd9 Ω cat*
JM109 / pSK98	*amyE::PmifM gfp-apdP(K128A)-flag-lacZd9 Ω cat*
JM109 / pSK99	*amyE::PmifM gfp-apdP(C129A)-flag-lacZd9 Ω cat*
JM109 / pSK100	*amyE::PmifM gfp-apdP(I130A)-flag-lacZd9 Ω cat*
JM109 / pSK101	*amyE::PmifM gfp-apdP(P133A)-flag-lacZd9 Ω cat*
JM109 / pSK106	*amyE::PmifM gfp-apdP(A132S)-flag-lacZd9 Ω cat*
JM109 / pSK107	*amyE::PmifM gfp-apdP(R131A)-flag-lacZd9 Ω cat*
JM109 / pSK109	*amyE::PmifM gfp-apdP(S127A)-flag-lacZd9 Ω cat*

Supplementary Table S6: Template DNA for PURE system

Description	Figure	1st PCR			2nd PCR		
		Primer 1	Primer 2	PCR template	Primer 3	Primer 4	PCR template
gfp-apcA-flag	Fig. 2	PP1	PP2	pCH2105	PP3	PP2	1st PCR product
gfp-apcA(R98A)-flag	Fig. 2	PP1	PP2	pCH2107	PP3	PP2	1st PCR product
gfp-apdA-flag	Fig. 2	PP1	PP2	pCH2108	PP3	PP2	1st PCR product
gfp-apdA(R120A)-flag	Fig. 2	PP1	PP2	pCH2118	PP3	PP2	1st PCR product
gfp-apdP-flag	Fig. 2	PP1	PP2	pCH2120	PP3	PP2	1st PCR product
gfp-apdP(R131A)-flag	Fig. 2	PP1	PP2	pCH2122	PP3	PP2	1st PCR product
gfp-apcA	Fig. 5	PP1	PP2	pCH2140	PP3	PP2	1st PCR product
gfp-apdA	Fig. 5	PP1	PP2	pCH2143	PP3	PP2	1st PCR product
gfp-apdP	Fig. 5	PP1	PP2	pCH2141	PP3	PP2	1st PCR product
gfp-apcA(R98A)-flag	Fig. 6	PP4	PP2	pCH2140	PP3	PP5	1st PCR product
gfp-apcA-P106tr	Fig. 6	PP4	PP2	pCH2140	PP3	PP6	1st PCR product
gfp-apcA-P106Gtr	Fig. 6	PP4	PP2	pCH2140	PP3	PP7	1st PCR product
gfp-apdA-P122(CCACCC)	Fig. 6	PP4	PP2	pCH2143	PP3	PP8	1st PCR product
gfp-apdA-P122(CCCCCA)	Fig. 6	PP4	PP2	pCH2143	PP3	PP9	1st PCR product
gfp-apdA-P122(CCA)tr	Fig. 6	PP4	PP2	pCH2143	PP3	PP10	1st PCR product
gfp-apdA-P122(CCC)tr	Fig. 6	PP4	PP2	pCH2143	PP3	PP11	1st PCR product
gfp-apdP-P133(CCACCC)	Fig. 6	PP4	PP2	pCH2141	PP3	PP12	1st PCR product
gfp-apdP-P133(CCCCCA)	Fig. 6	PP4	PP2	pCH2141	PP3	PP13	1st PCR product
gfp-apdP-P133(CCA)tr	Fig. 6	PP4	PP2	pCH2141	PP3	PP14	1st PCR product
gfp-apdP-P133(CCC)tr	Fig. 6	PP4	PP2	pCH2141	PP3	PP15	1st PCR product

Supplementary Table S7: Homologs of ApcA, ApdA and ApdP

Bacterial species	downstream gene	uORF	Protein ID	protein sequence
Rhodococcus erythropolis PR4	*yidC*	ApcA	BAH35453.1	MFCTLAVMQQRSTGTVTRSLLLLLLPFIALLAVPPLGSSGSAAVLGTAIAVFVVAALATSSKYELLQLCAVSATGPSDDERCLRGAFRRQSSPDAPGRPQPRAPGIGL
Rhodococcus fascians D188	*yidC*	ApcA	AMY51504.1	MLSIDNATRRLDSTAAFDVISRRAVSPWRAAVVTLVGVTAVLLVVFAVVDAQPAAQIAMFGALVAAALLVTGASADILVVVPPASTTAGSDGGRLRGAYRRQEQPDAPGRPMPRAPGSVHGPDRWLQFR
Amycolatopsis japonica	*yidC*	ApcA	WP_038523096.1	MERTYFGVRLKLMLALFLPELFVVLPVVGGINPLSLATALTASVAVANPLGLAAALTASLAAVLLALAVHFRLEPAAAAMRVRAISLRERAQLFLSLRDPDARGRVRPRAPSPGIVTA
Amycolatopsis mediterranei U32	*yidC*	ApcA	ADJ49378.1	MHTLLELAFPALTSPLALVAFASLAAASLLAVLLVGSTHRAELALWSAPVRSRATALRRRARRAESIRLRDPSRPGRSRPRAPGSRSTAA
Amycolatopsis orientalis HCCB10007	*yidC*	ApcA	AGM07380.1	MERTYFGVRLKLMLALFLPELFVVLPVVGGINPLSLATALTASVAVANPLGLAAALTASLAAVLLALAVHFRLEPAAAAMRVRAISLRERAQLFLSLRDPDARGRVRPRAPSPGIATA
Amycolatopsis vancoresmycina	*yidC*	ApcA	WP_004561741.1	MHTLLDLAFPALTSPTALVAFASLAAASLLVVLLVGSTHRAELALWSVPVRSRVTALRRRARRAESIRLRDPGAPGRSRPRAPGFRSTAA
Streptomyces ghanaensis ATCC 14672	*yidC*	ApcA	EFE69279.1	MRSRAFERMPGRRGGEGGRVIRSRTKPRSAVPAVLTVLTVLLPLLPVVLLALPEAGGLAAGVTLAATTAAGAALVVCALVAARCVPAVPPTRVRTALRDRDRRTAFLPQRDPDARGRTRPRAPGHALPATAAQGT
Streptomyces griseus subsp. griseus NBRC 13350	*yidC*	ApcA	BAG21513.1	MPYASSYGGIEPREGQEVVSVRDANALRRAFARLLRPAGLLVLFLTEVLLAEGGSLSAAVALAATAAAGSALLACSVISARCASPVPRTRVRTAIRDREKRTAFLPQRDPDARGRTRPRAPGHALLTAAA
Streptomyces lividans TK24	*yidC*	ApcA	AIJ15605.1	MTRERSVPRPLLVLLLVLLPVALVDVGSLSATVALAATAATAAVGSALAVCAVIAARCAPAVPRTRVRTAIRDRDLRTAFLPQRDPDAPGRRRPRAPGHALPATAA
Streptomyces sviceus ATCC 29083	*yidC*	ApcA	EDY54452.1	MAVMRDWTTPRPAALLLFLVLDLVLLDAGSFSAAVALAATAAAGSALAACSLIASRCAPAVPPTRVRTAIRDRARRTAFLSQRDPDAKGRPRPRAPGHALPATA
Streptomyces virginiae	*yidC*	ApcA	WP_051763714.1	MSFTLFARTSRALAVLLPLVLLGGLLGLFAGEGGLGAVVVALAATVAVGTEALAAAARLVRPVPPHRIRTAIRDREQRTAFLPQRDPDASGRSRPRAPGRLFPTAA
Amycolatopsis japonica	*secDF*	ApdA	AIG75889.1	MMVASRRTAKQAVLVLLAAVLTALGLFFALHSGGSASADESRGANATVESSVSKAVAPVRAAGRLAAEPALLGVHGHGDLPLFGTVPHGPASTTLLRLSELHDEAAPASRTANRLALGDRAPPFPVAV
Amycolatopsis orientalis HCCB10007	*secDF*	ApdA	AGM07482.1	MSMTVESRTTAAQAVPVLLAAVLTALGLFFALHSGGSASADEPRGANATVDSSVSKAVAPVRAAGRLAAEPALLGVHGHGDLPLFGTVPHGPASTTLLRLSELHDEATPASRTANRLAPGDRAPPFPVAV
Streptomyces coelicolor A3	*secDF*	ApdA	CAA22395.1	MRRRLASVLTVLLTVLLPLVPAWPAAGAHVGPSGPLAAAAATAVPHPALDLHADDGCTPVCAAQPRARHDQPAGRPTAPDQHPATTAHLGGGAAPCGHARTSFAPGPVPVSPGRASHDSGRAPPVSSGT
Streptomyces ghanaensis ATCC 14672	*secDF*	ApdA	EFE65575.1	MTTHRTGPVARLPLLAGVTFVTLAALLLTLVPGFTQEVRIAPFVSVSAPAPSMHPPDTRPYLDDAYVGAGTVLIRPQRDGGERTMSAGPPLLAPPHAPRVPPRPARPAPVAGHVPTDAAHVPSDLGRAPPASSSA
Streptomyces rimosus subsp. rimosus	*secDF*	ApdA	KUJ33630.1	MSGRARRALPGFLPRALAGVWAVLLVLAGTSGAAVAGAPGAALTGTPSTAGAASTPLGPGPLAVSSPAAAPSGPVDTAYRPSSAAPQALTAHSTPVPPRADARTTPAHPRADVRTAPDRPVARQSATAHVHAVLPHPRFPGLPGALPSVAPAPRHSPFGYGETVGPRQERAPPGTRYSPRSPRAPPFVRSS
Streptomyces scabiei 87.22	*secDF*	ApdA	CBG67800.1	MARGSSPAPRRGGLAPAALAVLLAVLTALFLPPSHGGAEHRPSSRVASAAGGHVTSAFEIIRADSGAQADDGHGAALLRSPRDVSGDRLPSPHTAALAAPRETAGPPRRGPTAAPAALPSPIPPPPTGRHQGRAPPPPPGT
Bartonella bacilliformis KC583	*secDF*	ApdP	ABM44462.1	MFFLHLHCRKRNFFFFAIGCFLLSNFMWLTDSSAFQDHLDEKQYTNDIINAYTYYLKHKYNQPNMMTATNKKFTKPHTFHHLSAVDSQDILILNVPPKPNTIIYVQLYSLQTQYLSNQRAPPLV
Brucella abortus 2308	*secDF*	ApdP	KFJ52591.1	MKTGSIPRLNSRESLGDLTKGRKKTEQDRQHGFMLFVTCVLSFTAFLFTVDFSSQAGDALNQPRGIAARQAEGSAAAPARQQSTPAPQPIRAILMEAIAAKIKTVWLGDGNAALLTKTAELHVSGNRSAEAAQIFQPLPALRGHFGSARAPPENA
Brucella melitensis bv. 1 str. 16M	*secDF*	ApdP	AIJ90026.1	MKTGSIPRLNSRESLGDLTKGRKKTEQDRQHGFMLFVTCVLSFTAFLFTVDFSSQAGDALNQPRGIAARQAEGSAAAPARQQSTPAPQPIRAILMEAIAAKIKTVWLGDGNAALLTKTAELHVSGNRSAEAAQIFQPLPALRGHFGSARAPPENA
Ochrobactrum anthropi ATCC 49188	*secDF*	ApdP	ABS14577.1	MKGMKNTEQHKRHGFMLFVTCVLSFTAFLFAVDFSTPDEDVARGHSGGIVARQVEGTPTTPVRQQSTPSPQHMRAILIEAIAAKIKAVYLDGTGAALPVAAVAFALPKSAAAKAALNFQRLAVLQRHFGSARAPPKSA
Agrobacterium radiobacter K84	*secDF*	ApdP	ACM25441.1	MNPFEFGAADNEENDERTHHAGTRVIASLFVAFFAYLGLALGGGINLAARADAEAFSSGRDSQPLYIAQRDPLRGMVVADRSALPKATWHDIGPITLAAIPSFGRLIDPWQPRDRGDVTRPEIFTFRAYLARAPPASFV
Ensifer adhaerens OV14	*secDF*	ApdP	WP_025425858.1	MDPVCGGNRGKFAGRLIAVLWLFCASLAMQLIVVGQGFSSRLSAEQQAGNFSAPDTVPSDHPAARQISRATPVSDLRFTAERKDGKSATGDGAVPMLLASVQFLAFEPSGEDLAFGRTPFADGLSNRGPQIRAPPRASGDC
Neorhizobium galegae bv. orientalis str. HAMBI 540	*secDF*	ApdP	CDN48227.1	MALFPQDKRMRDGREQESLLSRWPVFAAALLIAALFGLLSGVVKDVQFPRGVLSADKSSDTPHLTKREPLRALAAMDRKDGTGAHWPTGDGALEARAIEISFPIFVSAFASKPSSDPTAHPFWHGSLPRAPPARA
Rhizobium etli CFN 42	*secDF*	ApdP	WP_020920371	MGLDQHRLTSRIAASLLFAILAYIGLLFGGNLVISPAAALNNTLSSSGRDPQPQQLTARDAVRGVLAADRKLAPKQAAYDAGSPALAAPPVVDFAGRNMATAVPAPEAPLPAAVFRIYQPRAPPTVA
Rhizobium leguminosarum bv. trifolii CB782	*secDF*	ApdP	AHG44052.1	MELDQHHLTSRVAVSLLVAILAYLGLLLGGNLVISPAAAVNTLSSSGRDPQPPQLTARDAVRGVLATERRLAPKQAAHDGGSPAIAAAPVVDLAGWTISAAFPAFDASPHAAVSRTHQPRGPPAAA
Sinorhizobium fredii NGR234	*secDF*	ApdP	ACP24000	MDLLGGGSRNMQASRLLAVLWLFCASFAMQVITVGQSVSSRVGAQGGNVSAPDGGSPDRPAARQVSRAVALPELRIVGERPDGKSTLGGDPVAILAAEAATLPIYEDIRPLFFTRTEVAVGSRHDSERIRGPPAV
Sinorhizobium medicae WSM419	*secDF*	ApdP	ABR59039	MDLMRGGSRNMQASRLLAVLWLFCASLSMQVIAIGQSAASRAAGAPAGNVAQPDTGSSDRPVARQICRAVALPDLRFIGERADGKSCSGADPAAFVPFQAIALIAPEMAPSLPVAKTRIARLPSCQSKCIRAPPAAGAFL
Sinorhizobium meliloti 1021	*secDF*	ApdP	CAC45140.1	MHASRLLAVLWLFCASLSMQVIAVGQGAAGRAAGAPAGNVAQPDTGSSDRPVARQICRAVALPDLRFSGERADGKSMPGGDPAPCIQFQRVALAAPVASPSPSLPRAAIARAAPCQNECIRAPPPAAAIV
Alteromonas macleodii ATCC 27126	*secDF*	ApdP	AFS36256.1	MPLFMAWVLSCLILSASFQSGQINNEVEVSQQSPLAQFNYAGKSYSLRFTGTKLENSDDQGDGPEAIVELTLGFIALAIVSCLGMANVVPRARKHYRQPSRGPPLSLS
